# Supplementary material for: Vitamin D induces SIRT1 activation through K610 deacetylation in colon cancer
Source: eLife. 2023 Aug 2;12:RP86913. doi: 10.7554/eLife.86913 (PMC10396337; doi:10.7554/eLife.86913)
Supplement: Figure 1—source data 1. [file elife-86913-fig1-data1.zip › Fig1_source data/Fig 1B_raw TIFFand PDF/Fig.1B_raw data_pdf.pdf]

Figure 1B

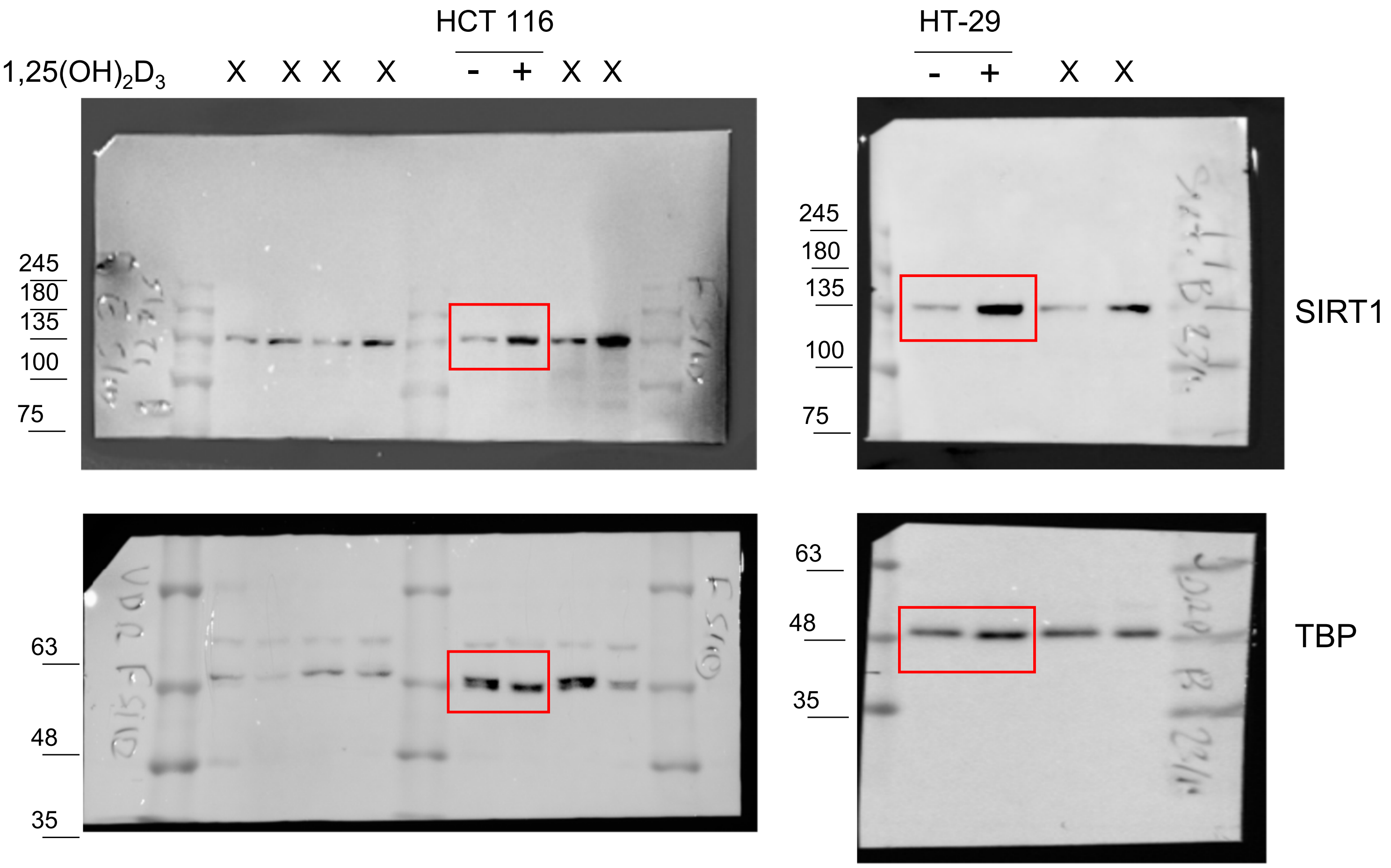
HCT 116
HT-29
1,25(OH)$_2$D$_3$
X X X X - + X X
- + X X
245
180
135
100
75
SIRT1
63
48
35
TBP

Figure 1G

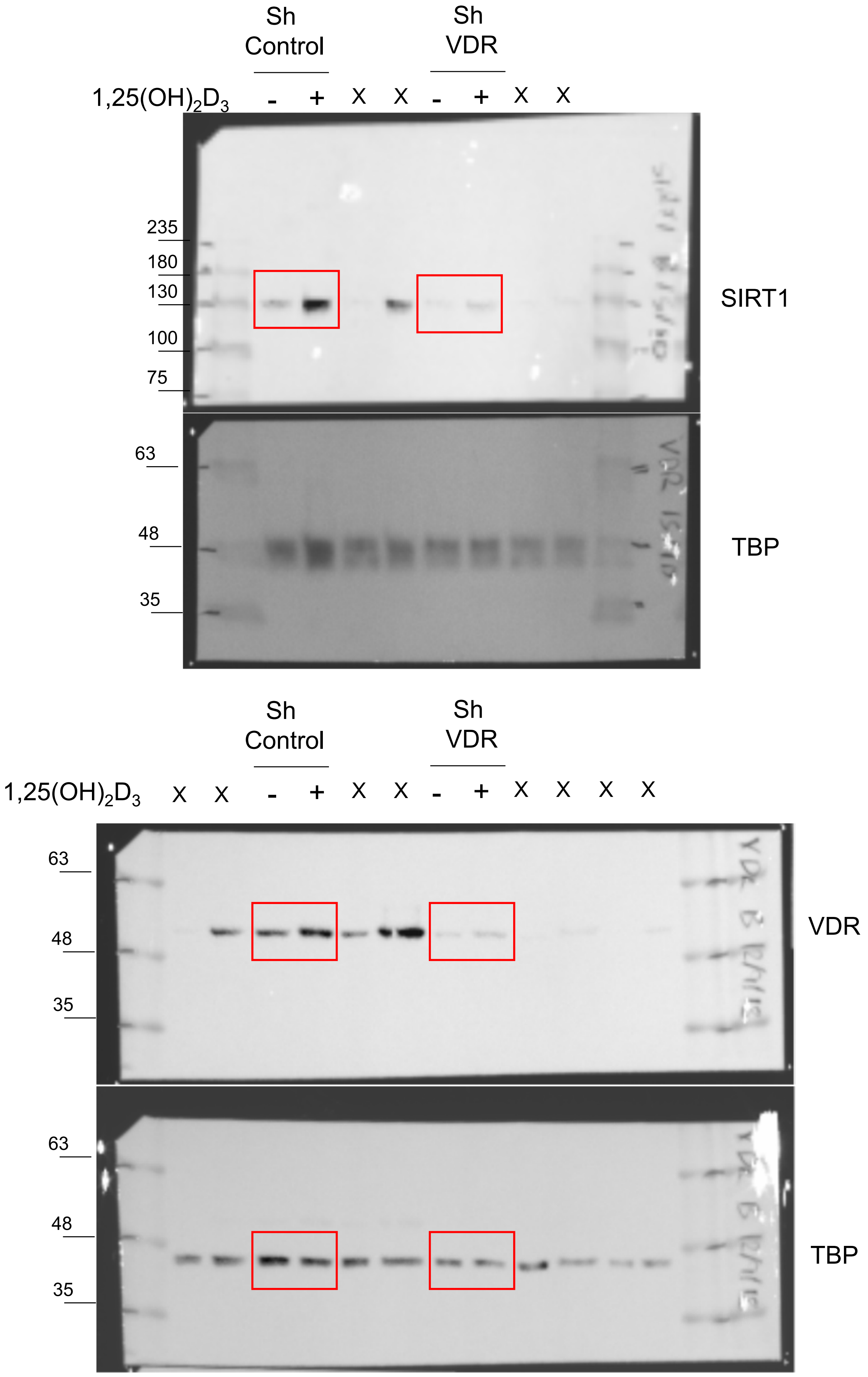
Sh Control
Sh VDR
1,25(OH)$_2$D$_3$ - + X X - + X X
235
180
130
100
75
SIRT1
63
48
35
TBP
Sh Control
Sh VDR
1,25(OH)$_2$D$_3$ X X - + X X - + X X X X
63
48
35
VDR
63
48
35
TBP

Figure 1H

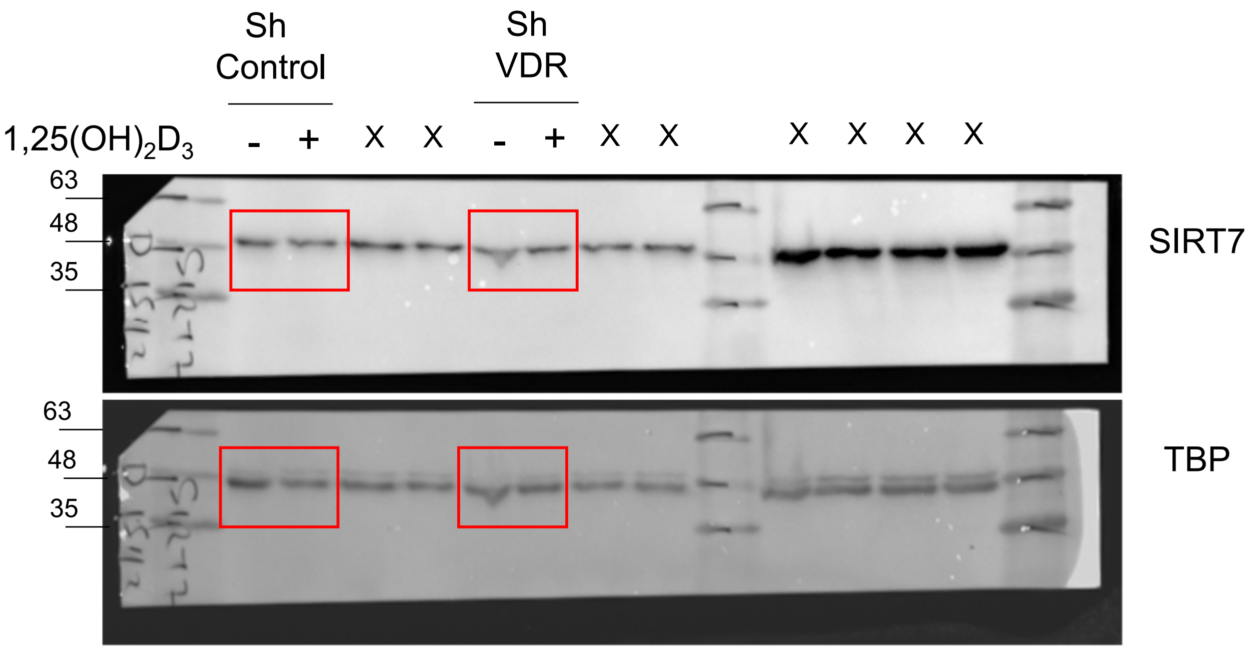

Sh
Control
Sh
VDR
1,25(OH)2D3
-
+
X
X
-
+
X
X
X
X
X
X
63
48
35
SIRT7
63
48
35
TBP

Figure 2C

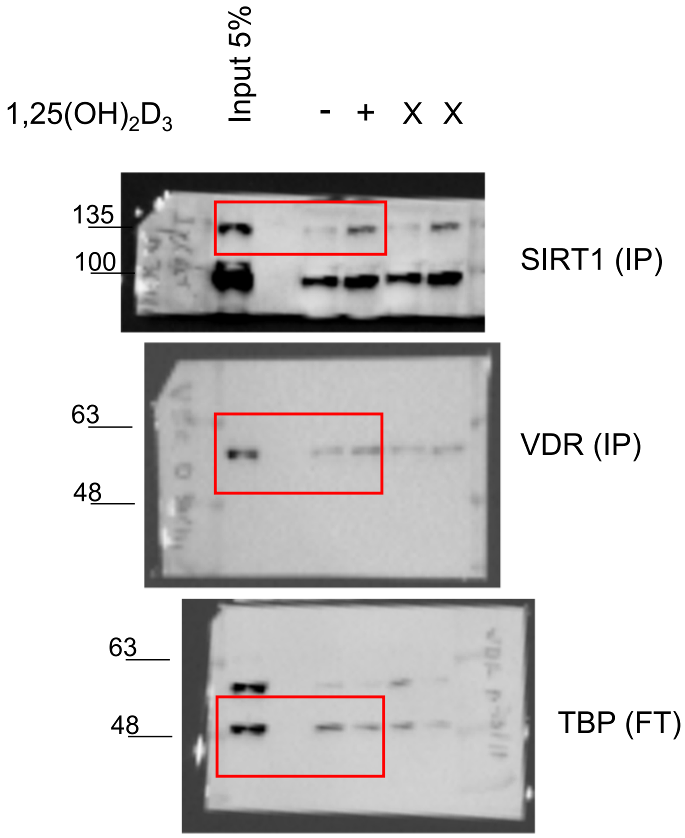

Figure 2D

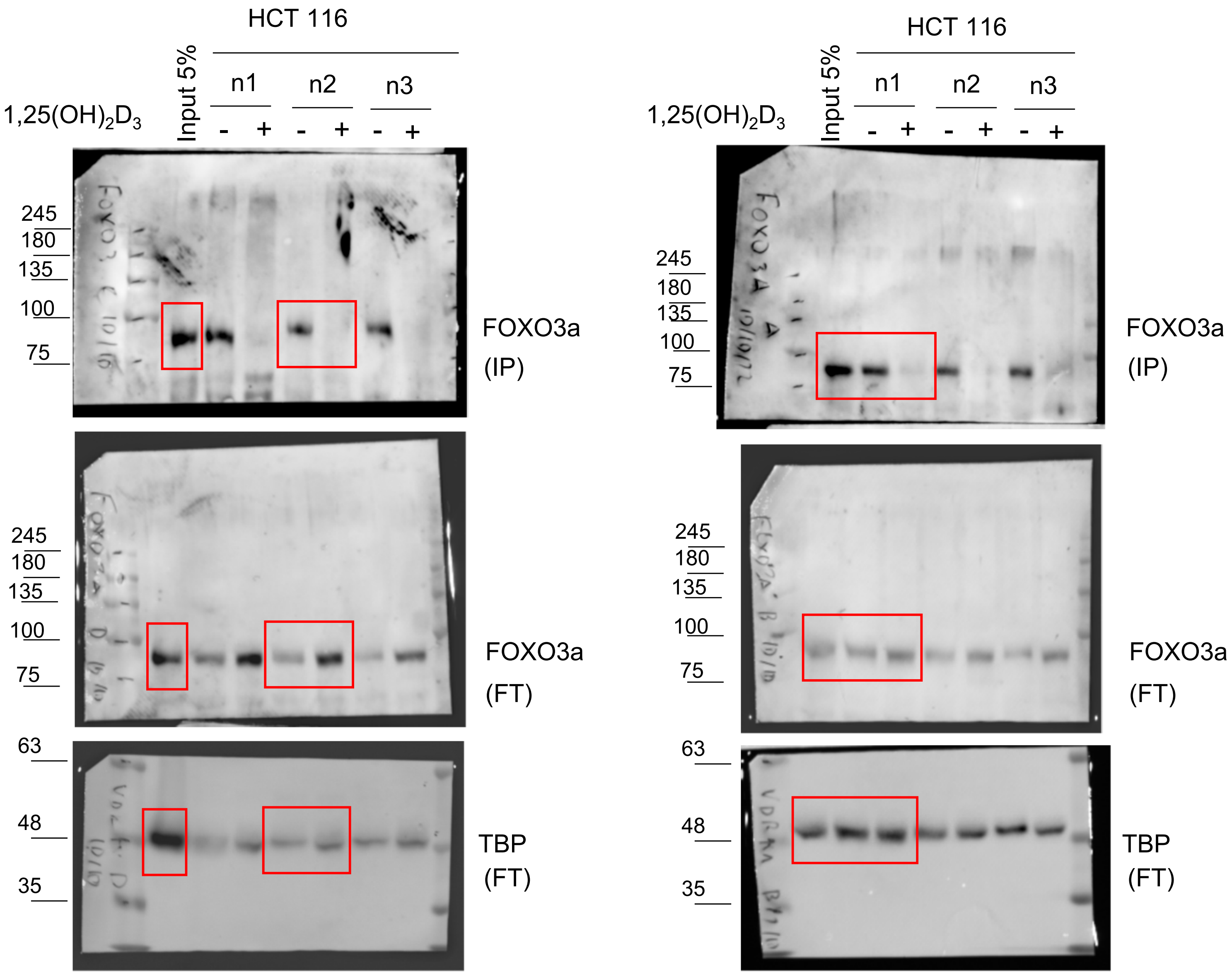
HCT 116
Input 5%
n1
n2
n3
1,25(OH)2D3
- + - + - +
245
180
135
100
75
FOXO3a
(IP)
FOXO3a
(FT)
63
48
35
TBP
(FT)
HCT 116
Input 5%
n1
n2
n3
1,25(OH)2D3
- + - + - +
245
180
135
100
75
FOXO3a
(IP)
FOXO3a
(FT)
63
48
35
TBP
(FT)

Figure 2E

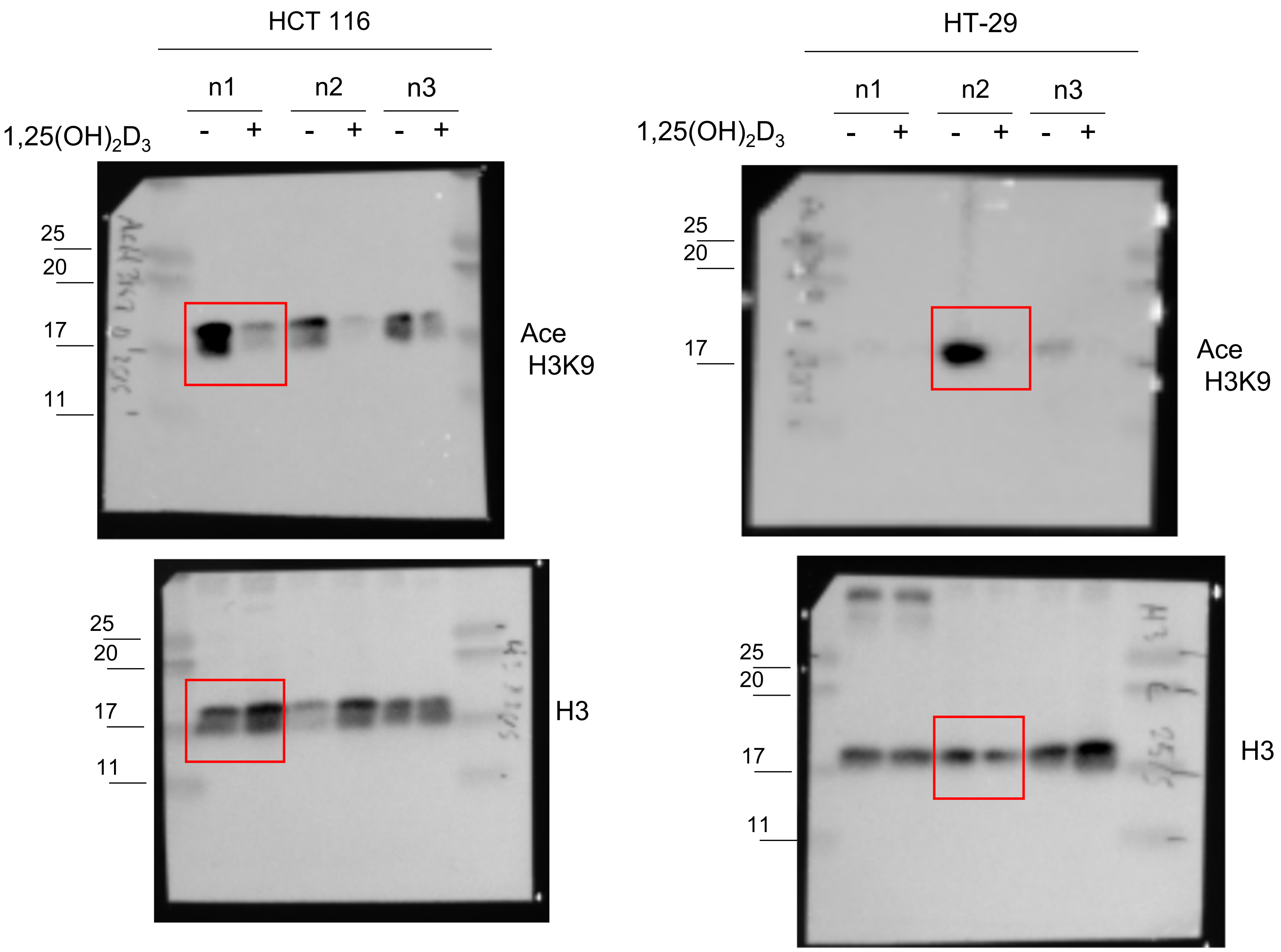
HCT 116
n1
n2
n3
1,25(OH)2D3
- + - + - +
25
20
17
11
Ace H3K9
H3
HT-29
n1
n2
n3
1,25(OH)2D3
- + - + - +
25
20
17
11
Ace H3K9
H3

Figure 2F

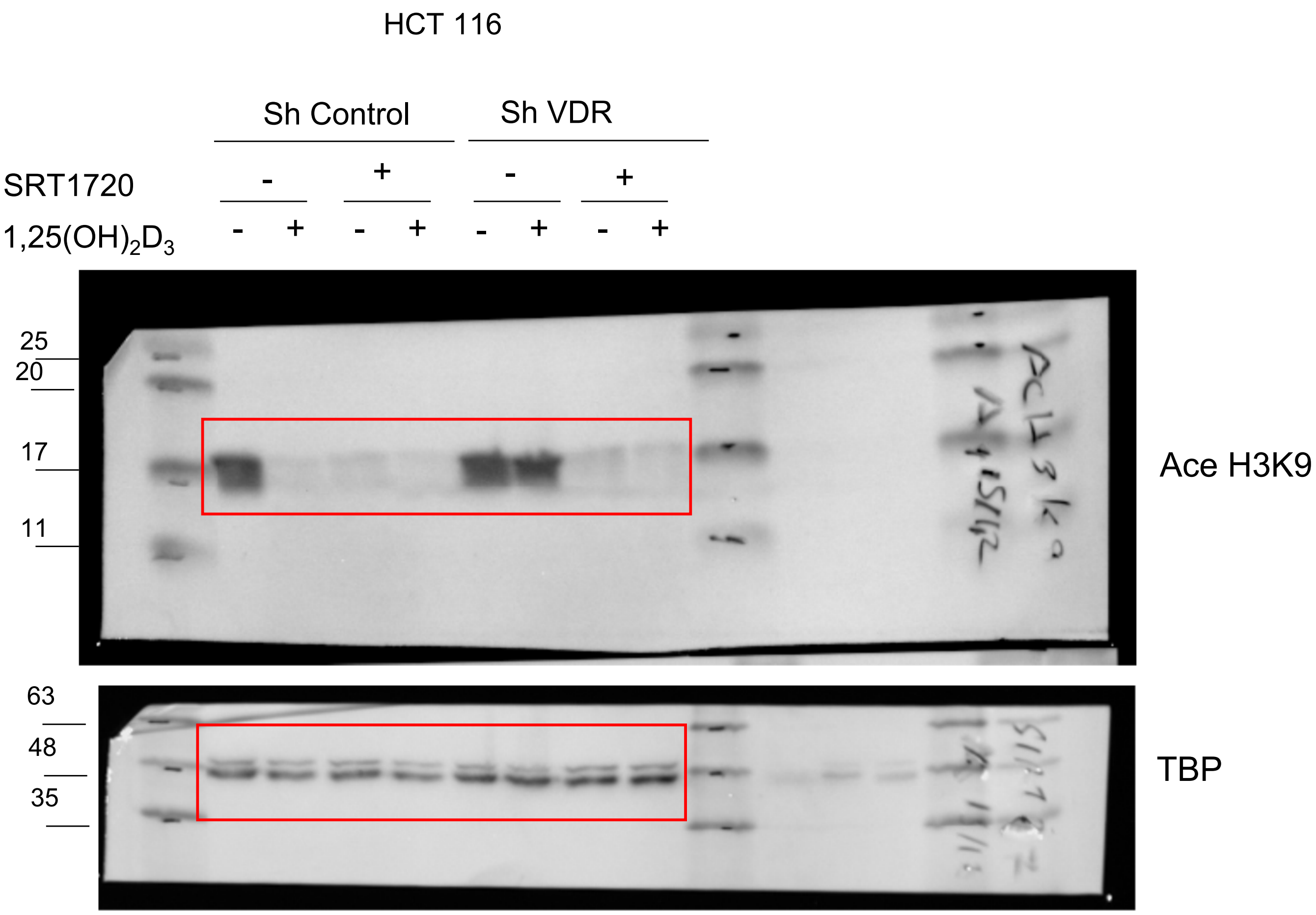

HCT 116
Sh Control
Sh VDR
SRT1720
- +
- +
1,25(OH)2D3
- + - + - + - +
25
20
17
11
Ace H3K9
63
48
35
TBP

Figure 2G

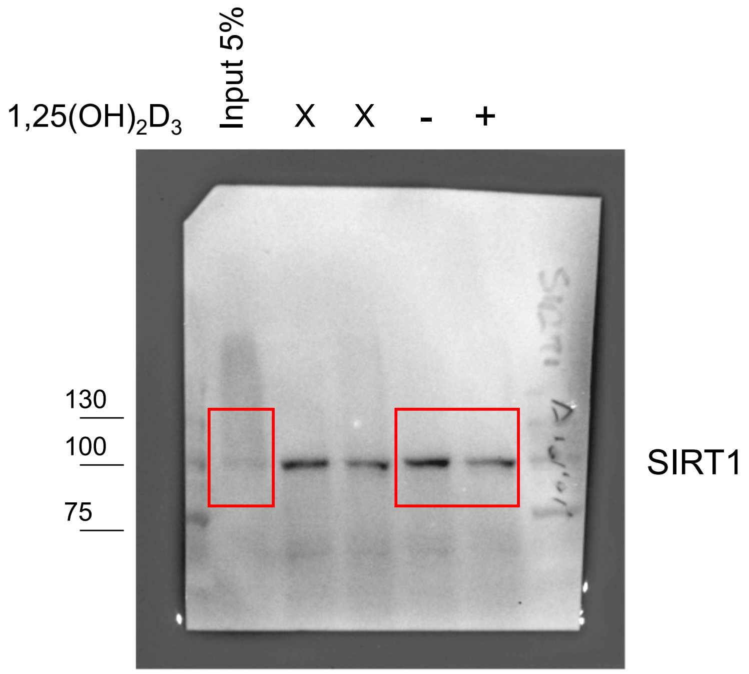

Figure 3F

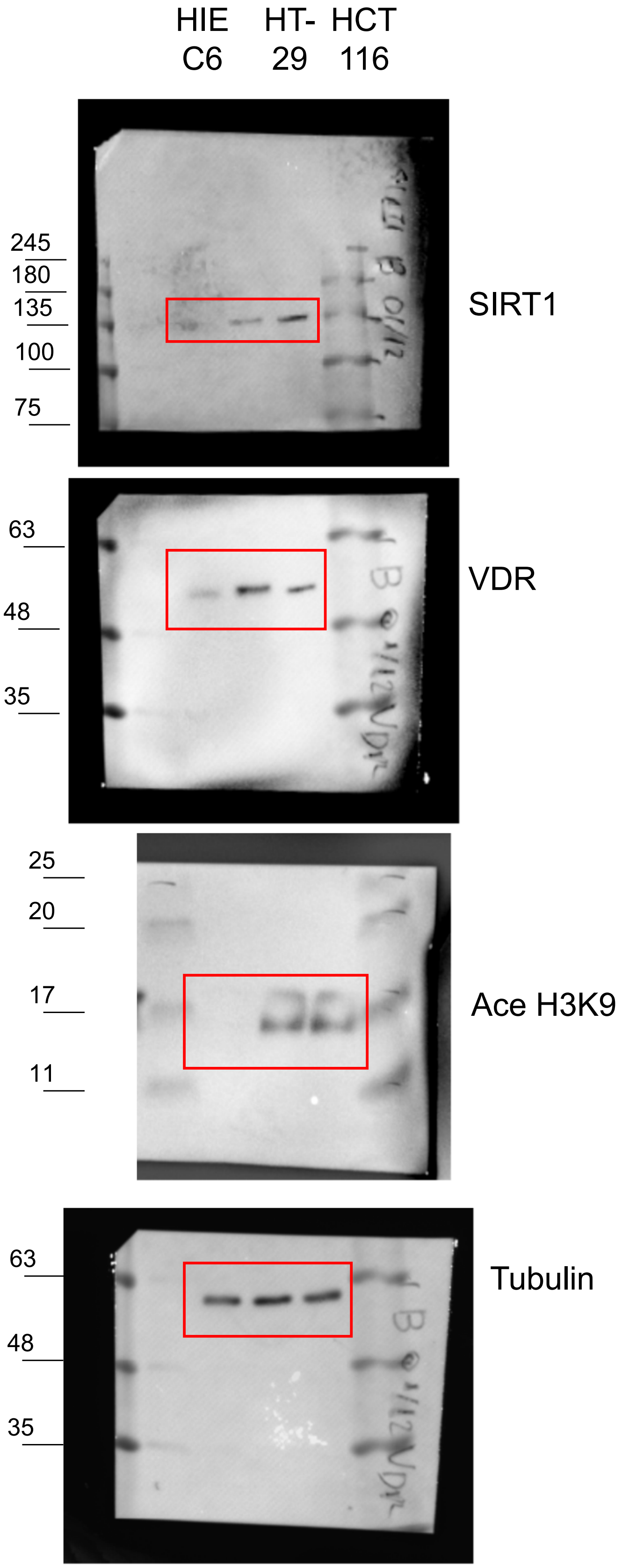

HIE C6
HT-29
HCT 116
245
180
135
100
75
SIRT1
63
48
35
VDR
25
20
17
11
Ace H3K9
63
48
35
Tubulin

Figure 3G

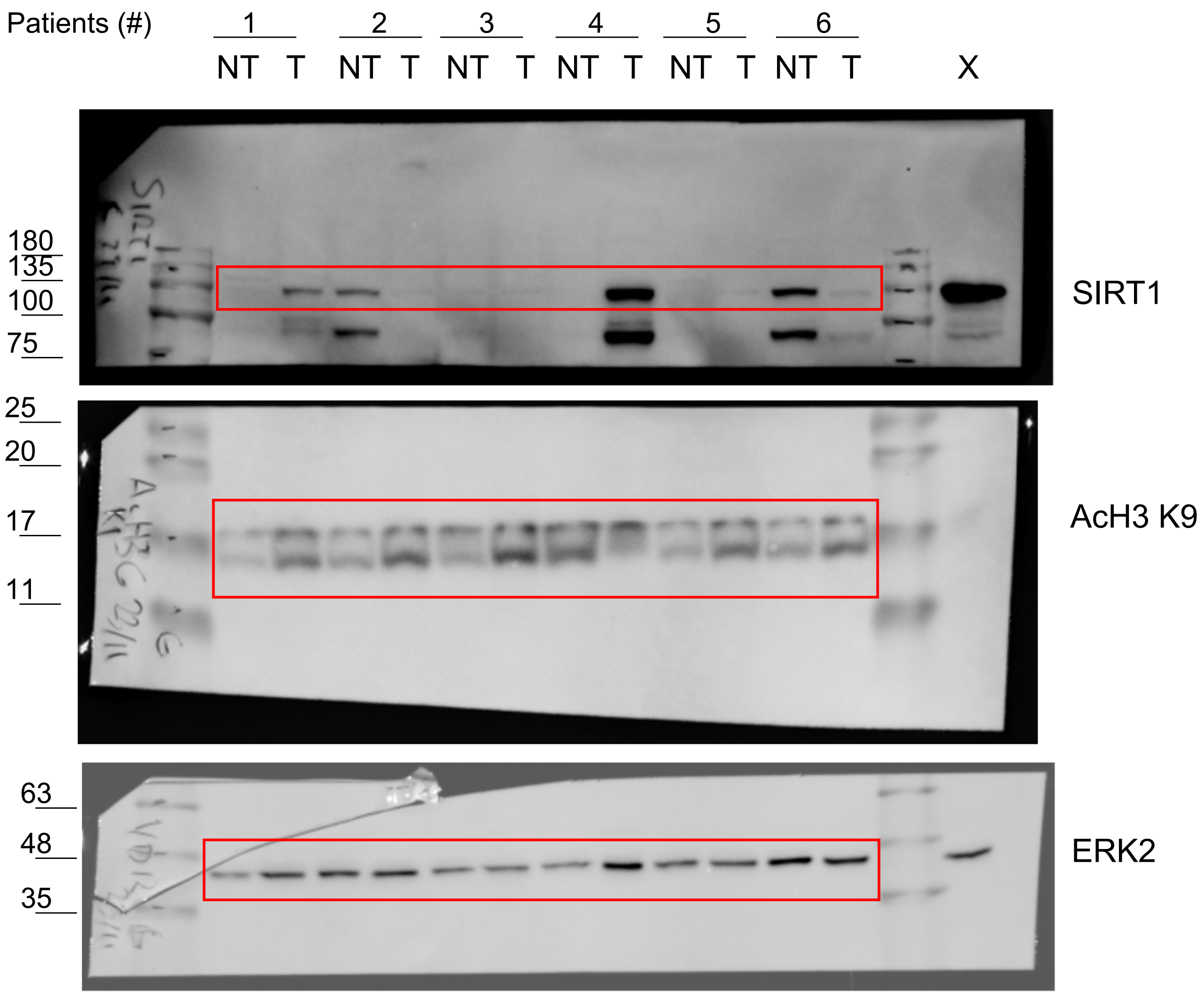

Patients (#)
1
2
3
4
5
6
NT T NT T NT T NT T NT T NT T X
180
135
100
75
SIRT1
25
20
17
11
AcH3 K9
63
48
35
ERK2

Figue 3H

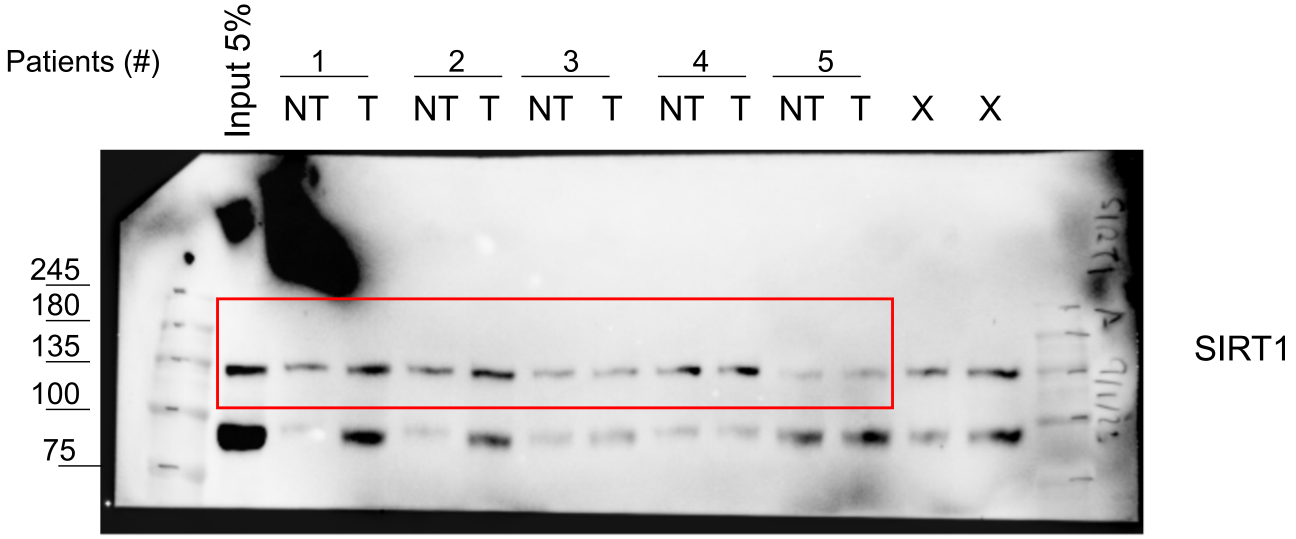

Patients (#)
Input 5%
1
2
3
4
5
NT T NT T NT T NT T NT T X X
245
180
135
100
75
SIRT1

Figure 3I

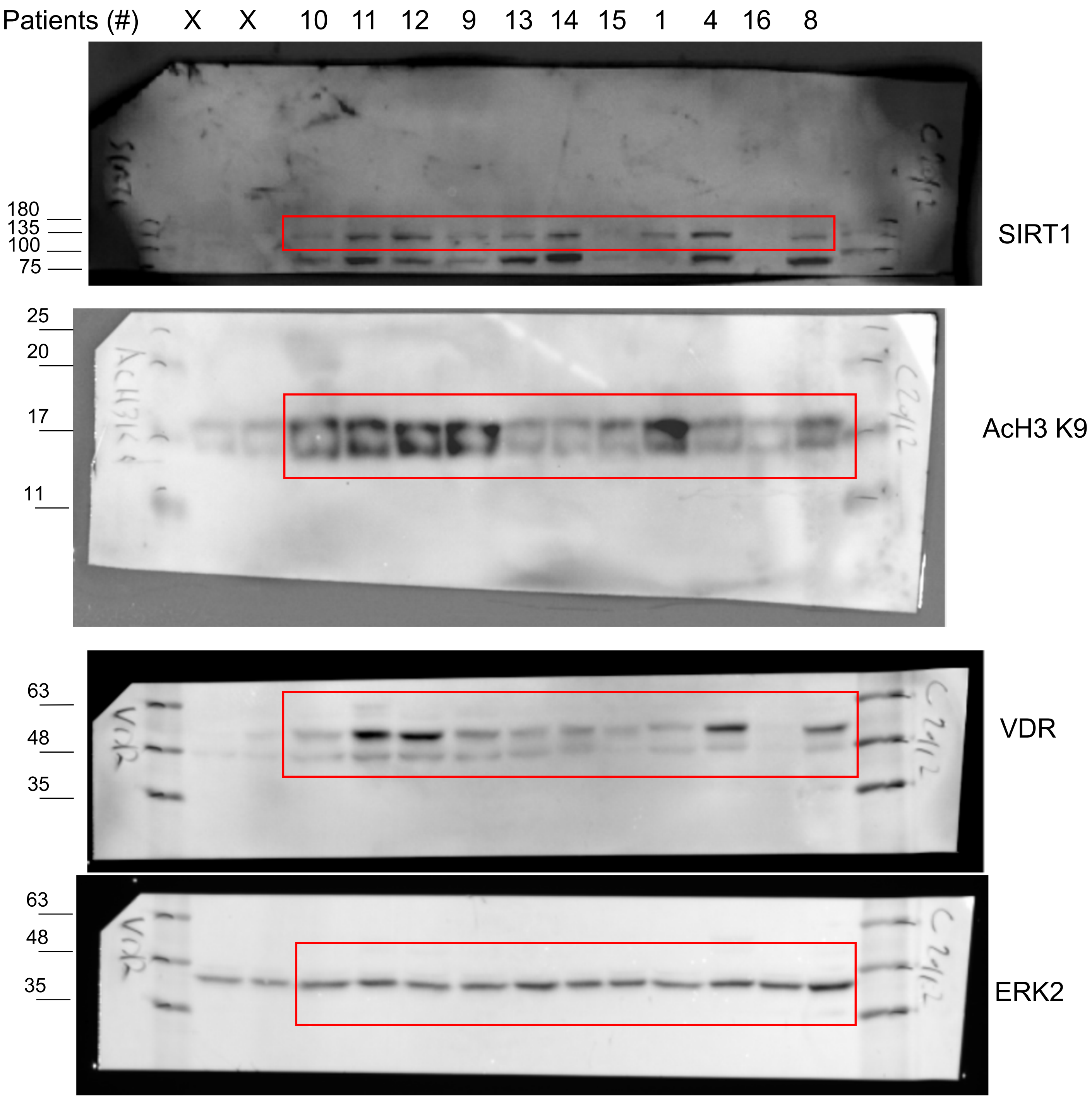
Patients (#) X X 10 11 12 9 13 14 15 1 4 16 8
180
135
100
75
SIRT1
25
20
17
11
AcH3 K9
63
48
35
VDR
63
48
35
ERK2

Figure 3J

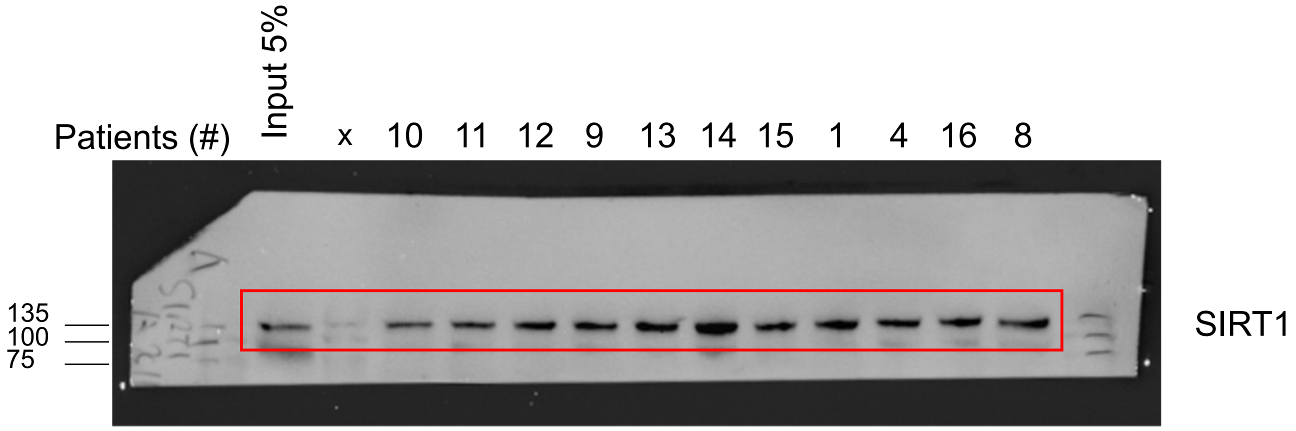

Figure 4A

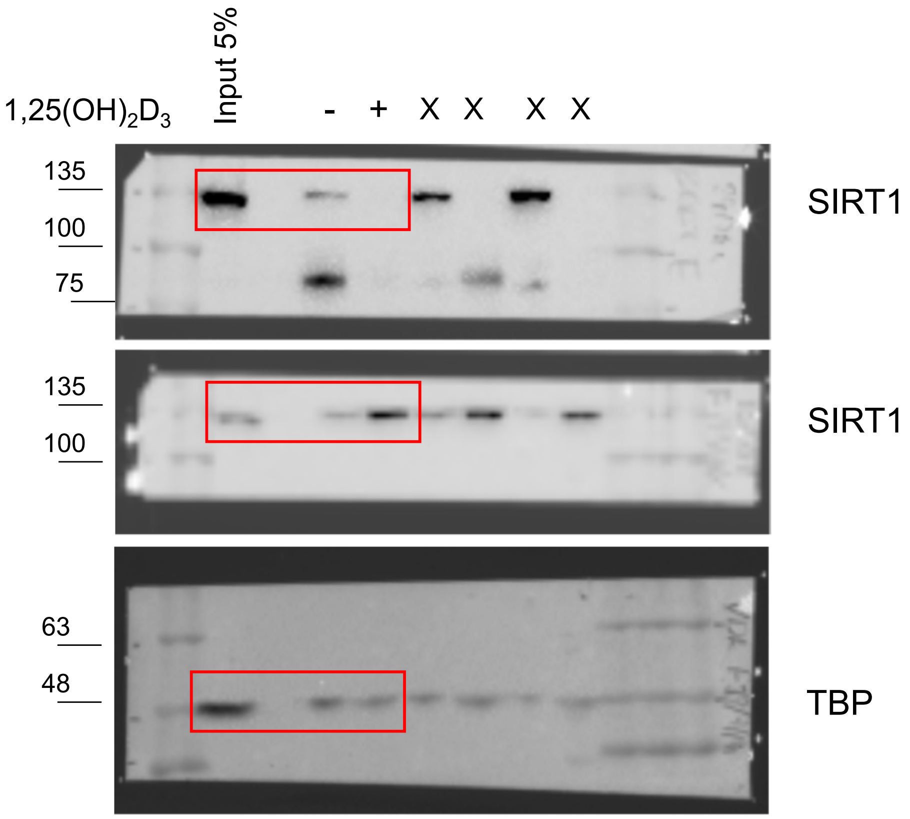

Figure 4B

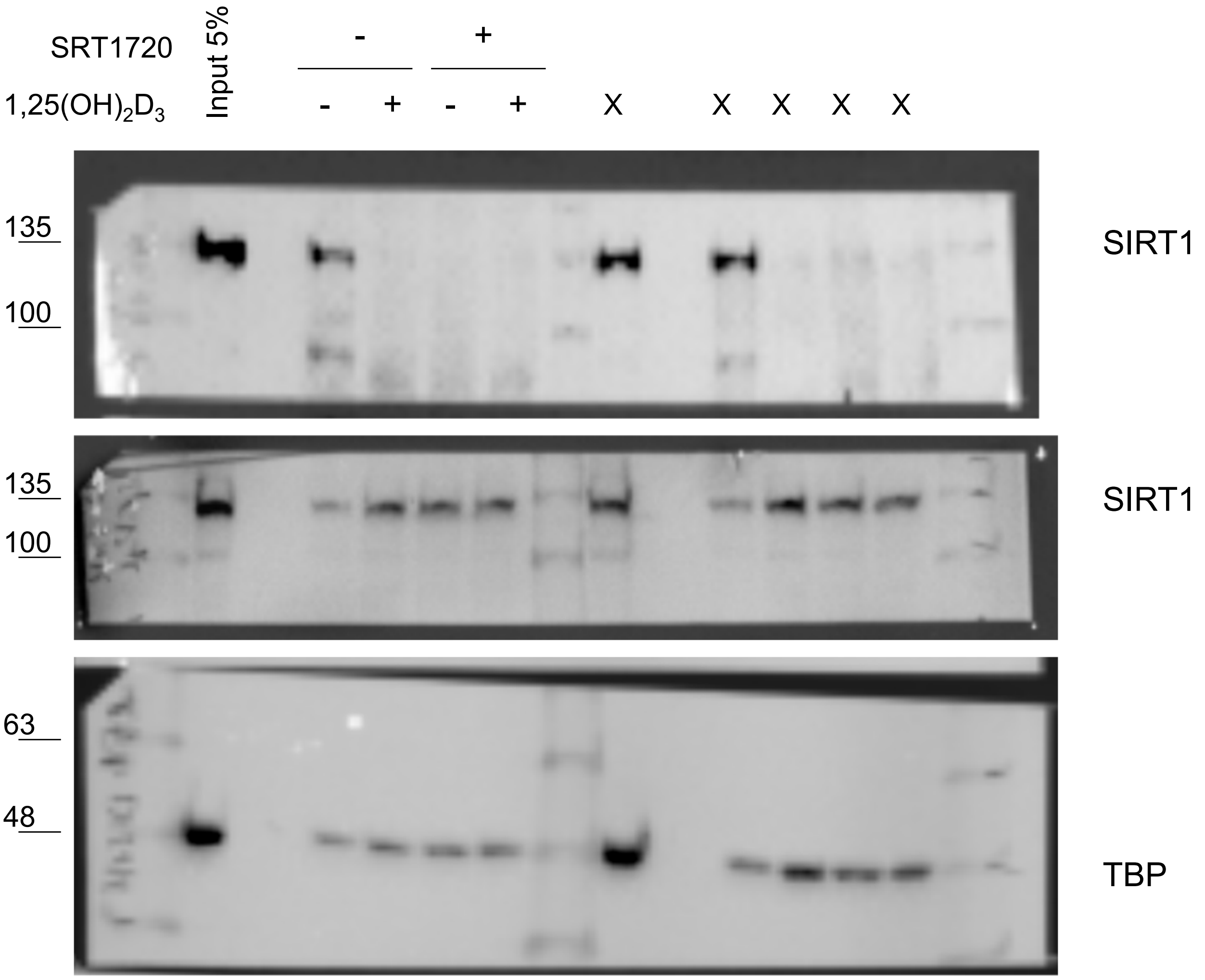
SRT1720
Input 5%
-
+
1,25(OH)2D3
- + - + X X X X X
135
100
SIRT1
135
100
SIRT1
63
48
TBP

Figure 4C

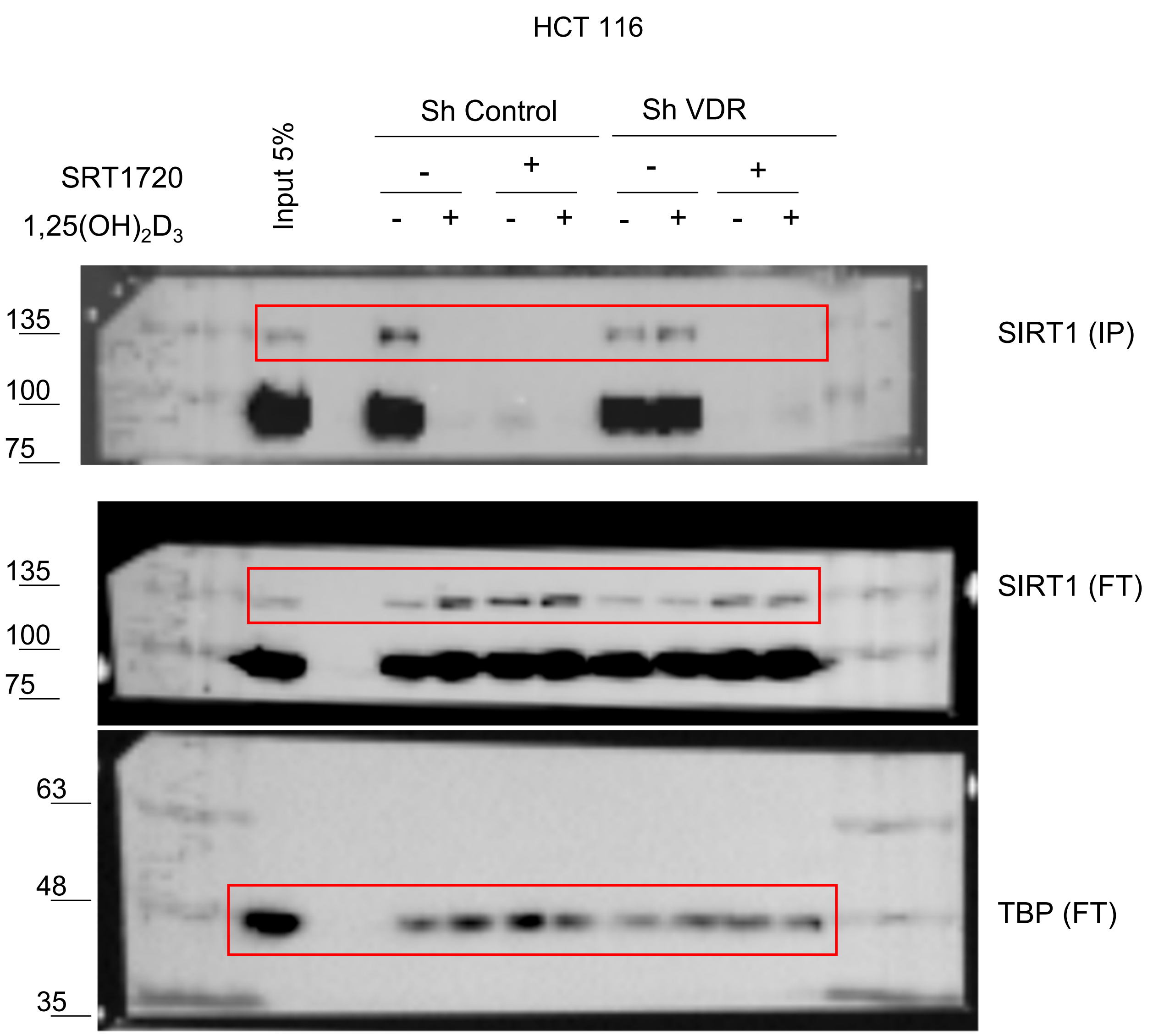

HCT 116
Sh Control
Sh VDR
SRT1720
1,25(OH)2D3
Input 5%
- + - + - + - +
135
100
75
SIRT1 (IP)
135
100
75
SIRT1 (FT)
63
48
35
TBP (FT)

low exposure

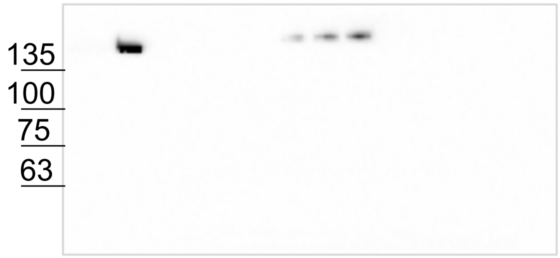

High exposure

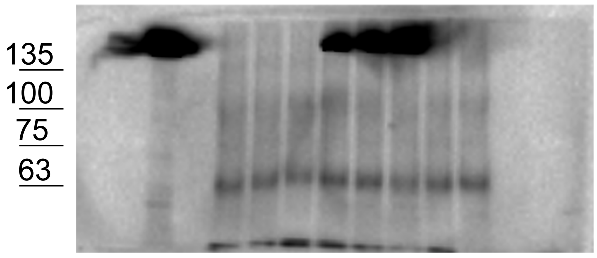

Suppl Fig 1

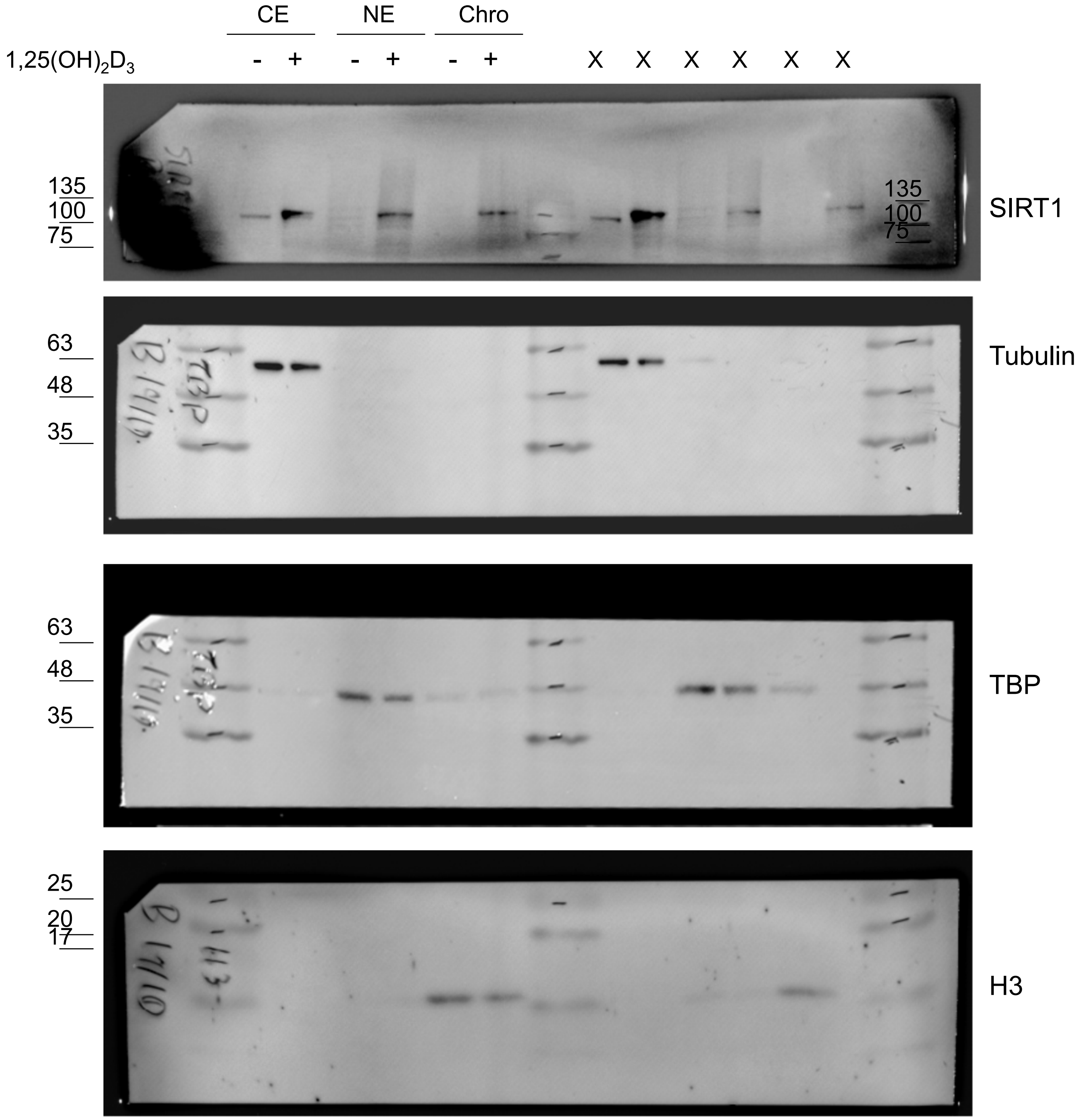
CE
NE
Chro
1,25(OH)2D3
- + - + - +
X X X X X X
135
100
75
SIRT1
63
48
35
Tubulin
63
48
35
TBP
25
20
17
H3
