# Supplementary figures and images for: Vitamin D induces SIRT1 activation through K610 deacetylation in colon cancer
Source: eLife. 2023 Aug 2;12:RP86913. doi: 10.7554/eLife.86913 (PMC10396337; doi:10.7554/eLife.86913)

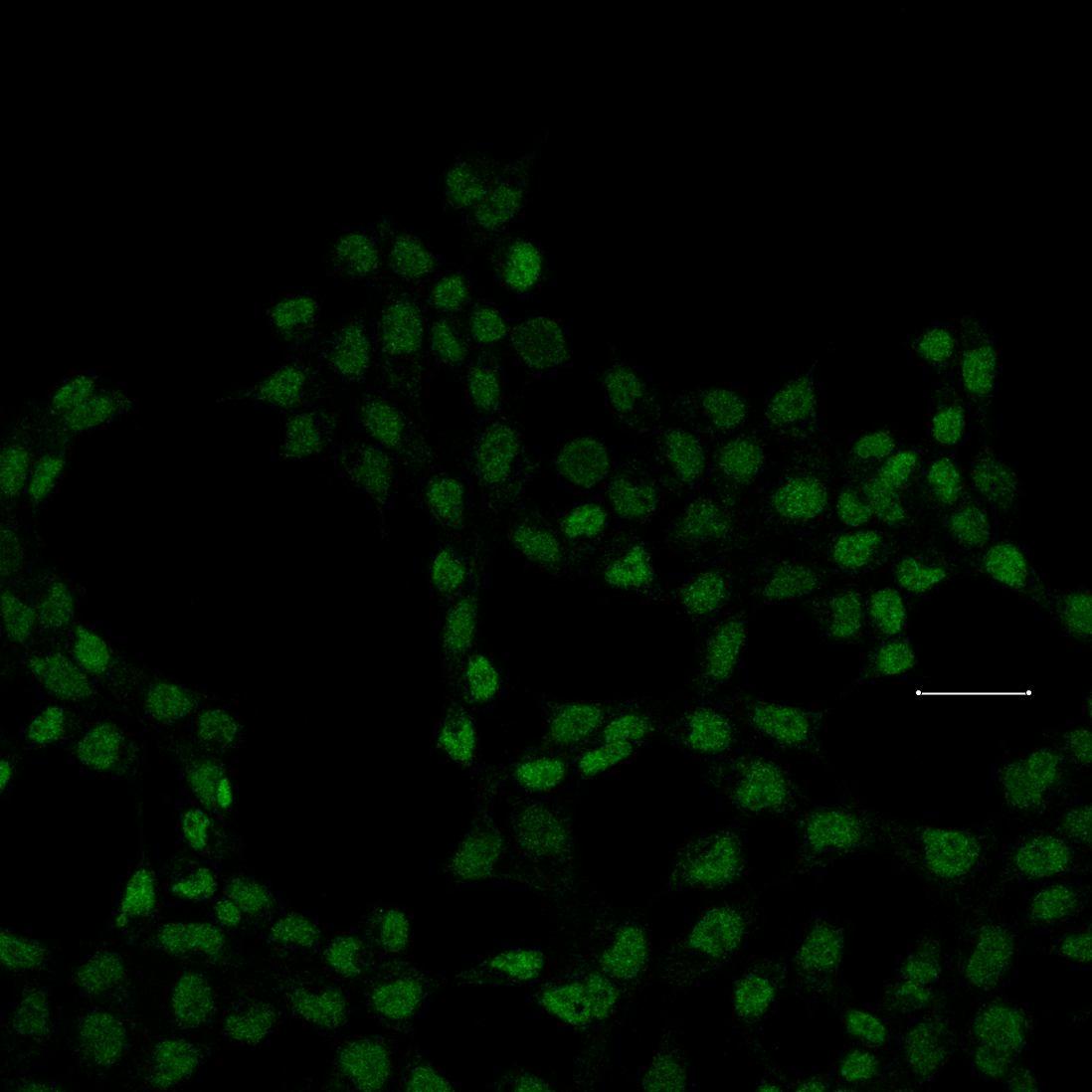

Supplement: Figure 1—source data 1. [file elife-86913-fig1-data1.zip › Fig1_source data/FIG 1A_raw TIFF/HCT 116 -VIt D.tif]

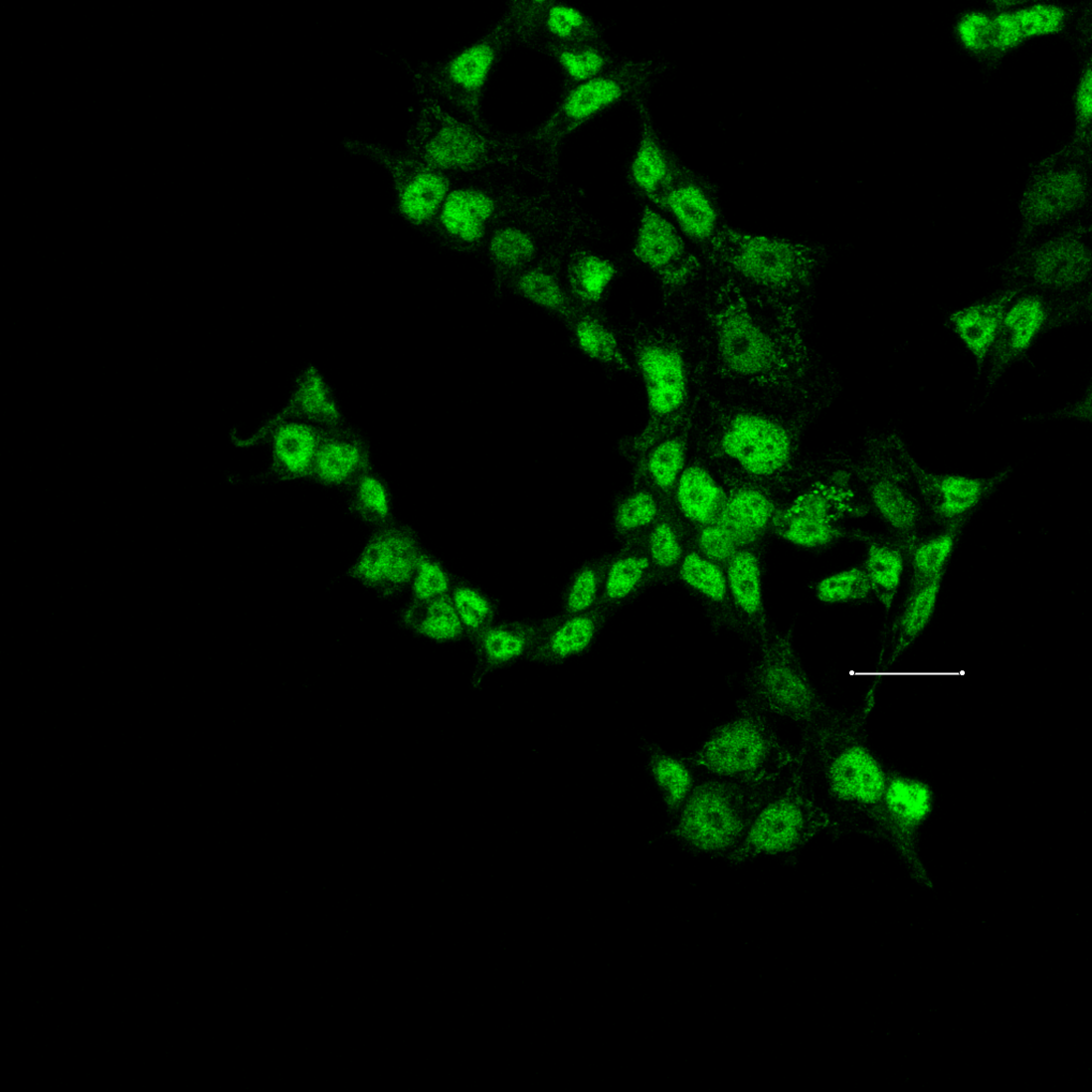

Supplement: Figure 1—source data 1. [file elife-86913-fig1-data1.zip › Fig1_source data/FIG 1A_raw TIFF/HCT116 +Vit D.tif]

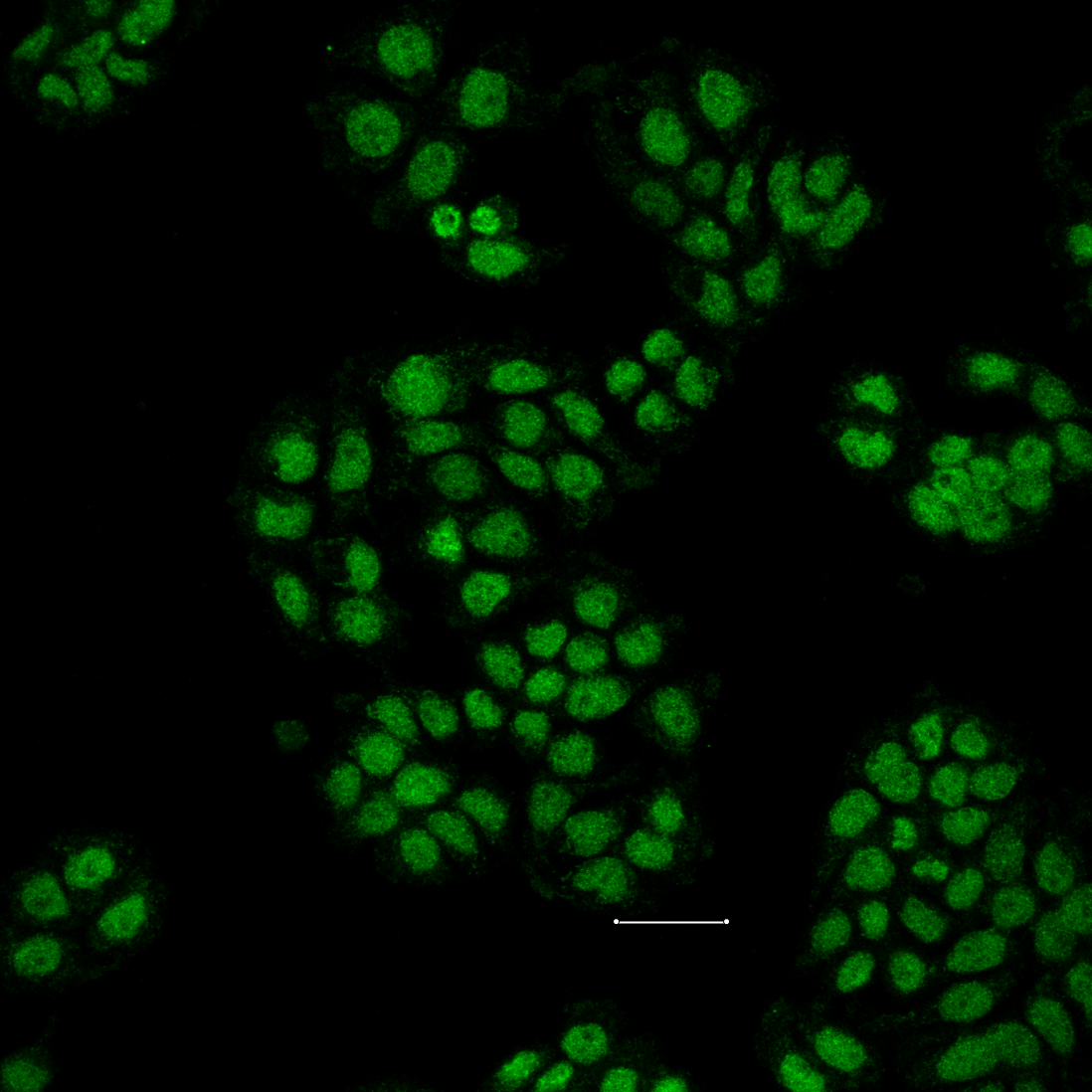

Supplement: Figure 1—source data 1. [file elife-86913-fig1-data1.zip › Fig1_source data/FIG 1A_raw TIFF/HT-29 +VItD .tif]

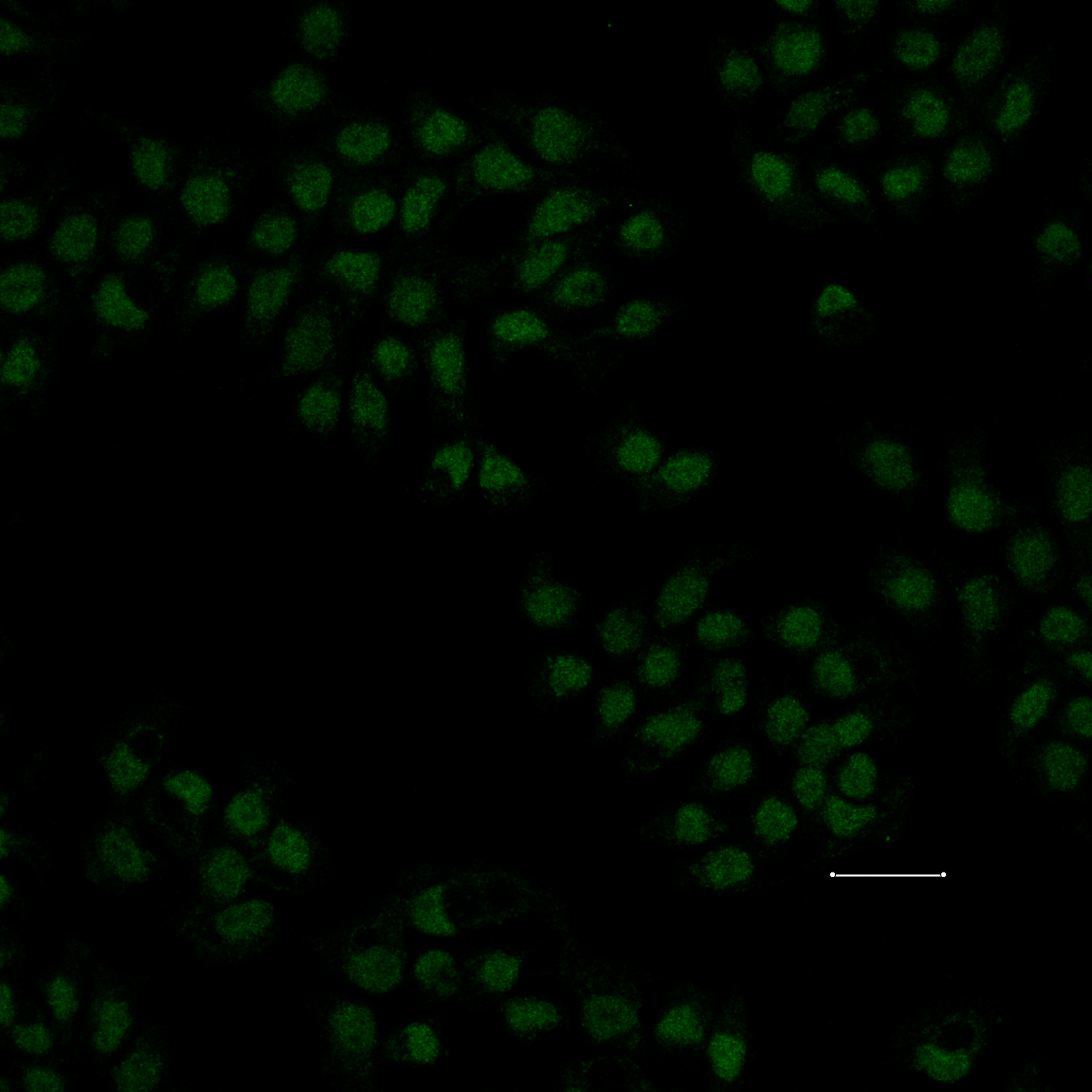

Supplement: Figure 1—source data 1. [file elife-86913-fig1-data1.zip › Fig1_source data/FIG 1A_raw TIFF/HT-29 -Vit D.tif]

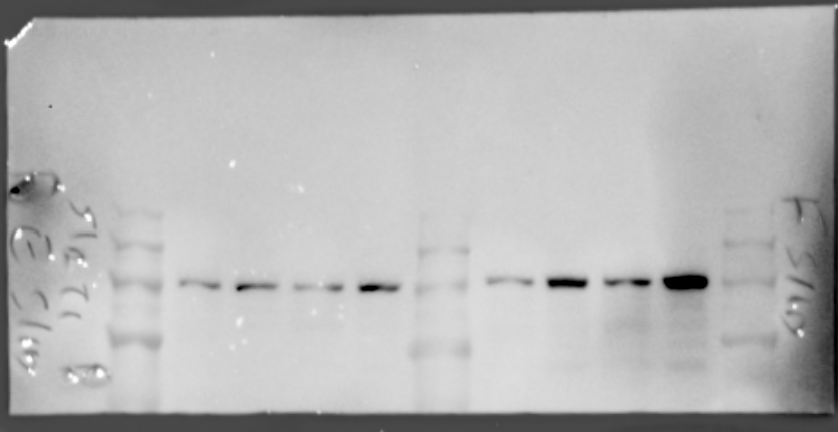

Supplement: Figure 1—source data 1. [file elife-86913-fig1-data1.zip › Fig1_source data/Fig 1B_raw TIFFand PDF/Figure 1B_scan_HCT116_SIRT1.tif]

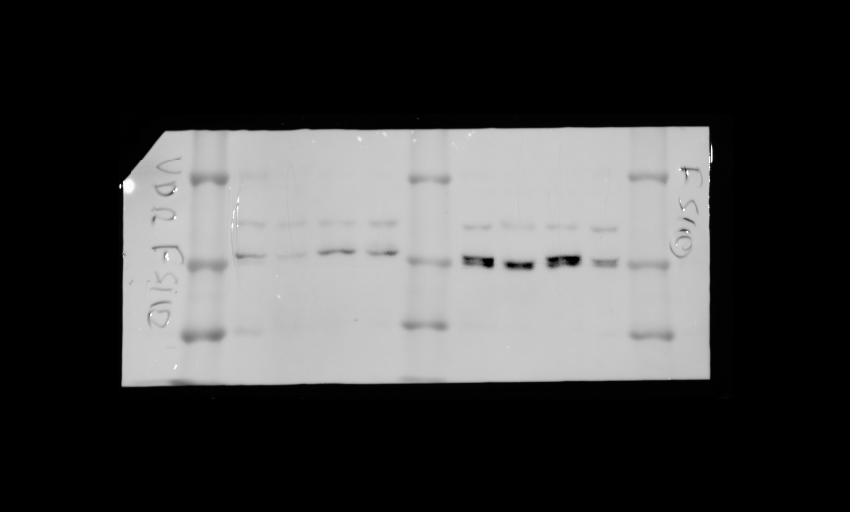

Supplement: Figure 1—source data 1. [file elife-86913-fig1-data1.zip › Fig1_source data/Fig 1B_raw TIFFand PDF/Figure 1B_scan_HCT116_TBP.tif]

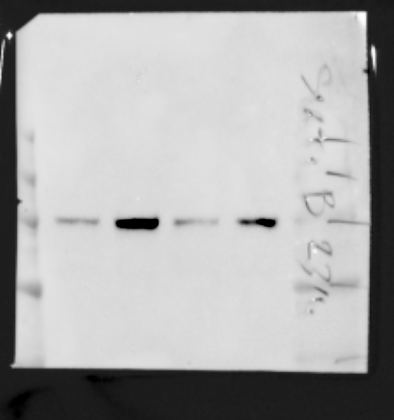

Supplement: Figure 1—source data 1. [file elife-86913-fig1-data1.zip › Fig1_source data/Fig 1B_raw TIFFand PDF/Figure 1B_scan_HT29_SIRT1.tif]

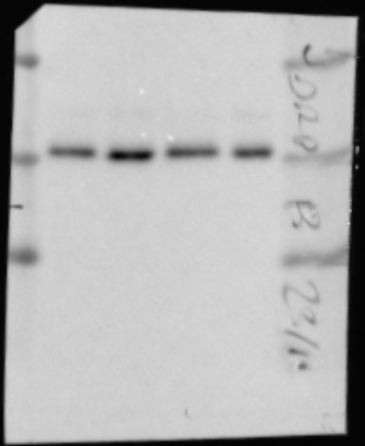

Supplement: Figure 1—source data 1. [file elife-86913-fig1-data1.zip › Fig1_source data/Fig 1B_raw TIFFand PDF/Figure 1B_scan_HT29_TBP.tif]

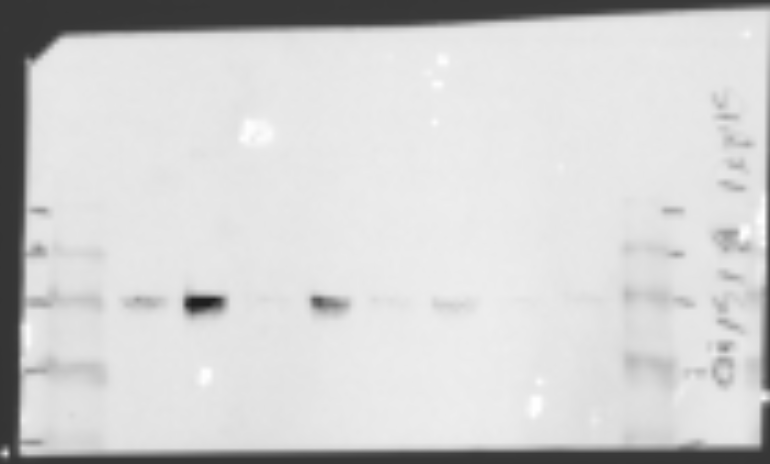

Supplement: Figure 1—source data 1. [file elife-86913-fig1-data1.zip › Fig1_source data/Fig 1G_raw TIFFand PDF/Figure 1G_scan_SIRT1.tif]

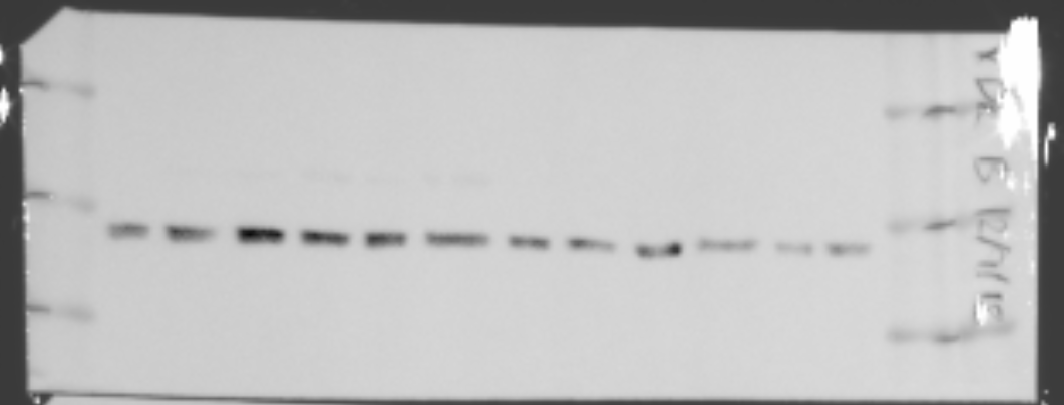

Supplement: Figure 1—source data 1. [file elife-86913-fig1-data1.zip › Fig1_source data/Fig 1G_raw TIFFand PDF/Figure 1G_scan_tbp.tif]

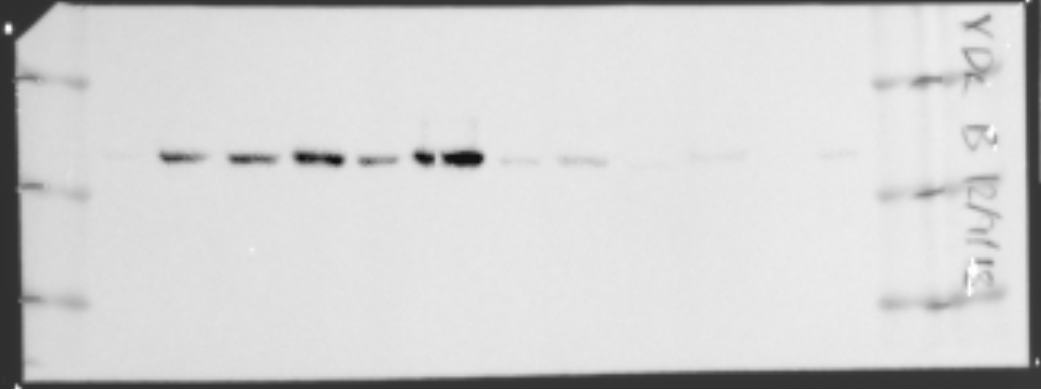

Supplement: Figure 1—source data 1. [file elife-86913-fig1-data1.zip › Fig1_source data/Fig 1G_raw TIFFand PDF/Figure 1G_scan_VDR.tif]

Figure 1G

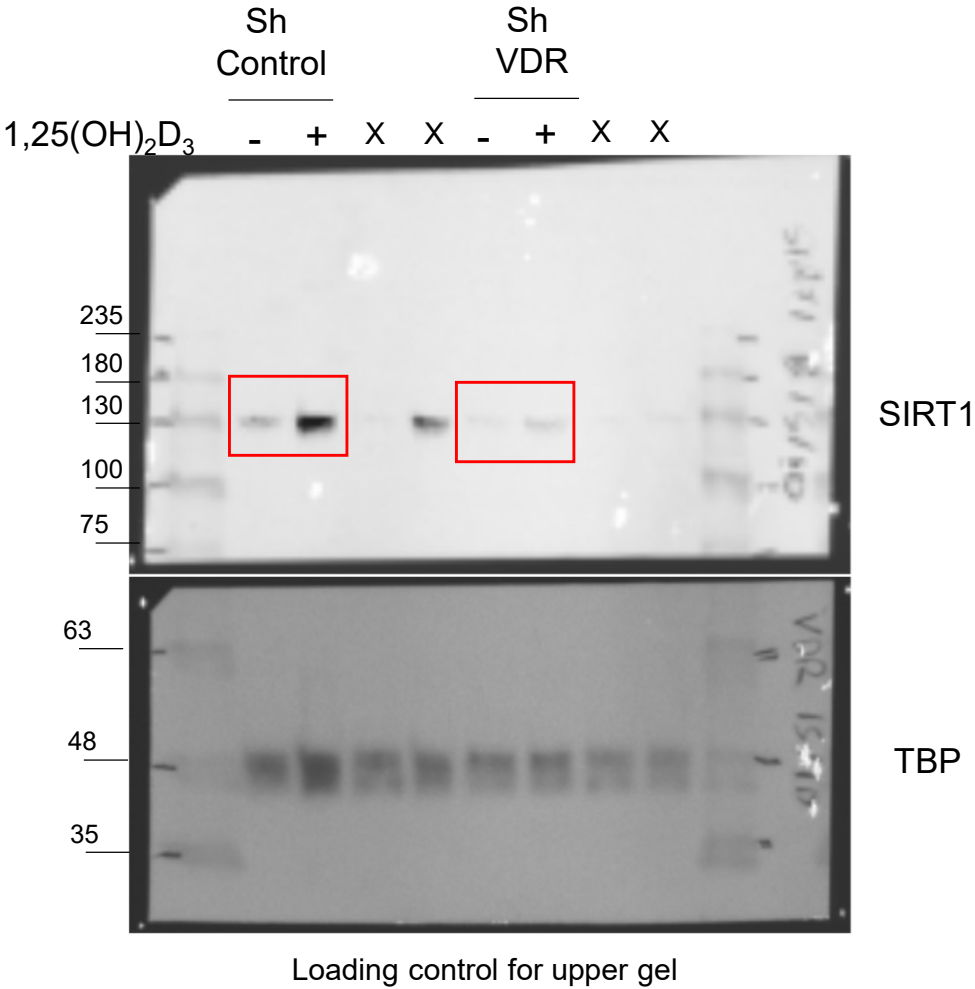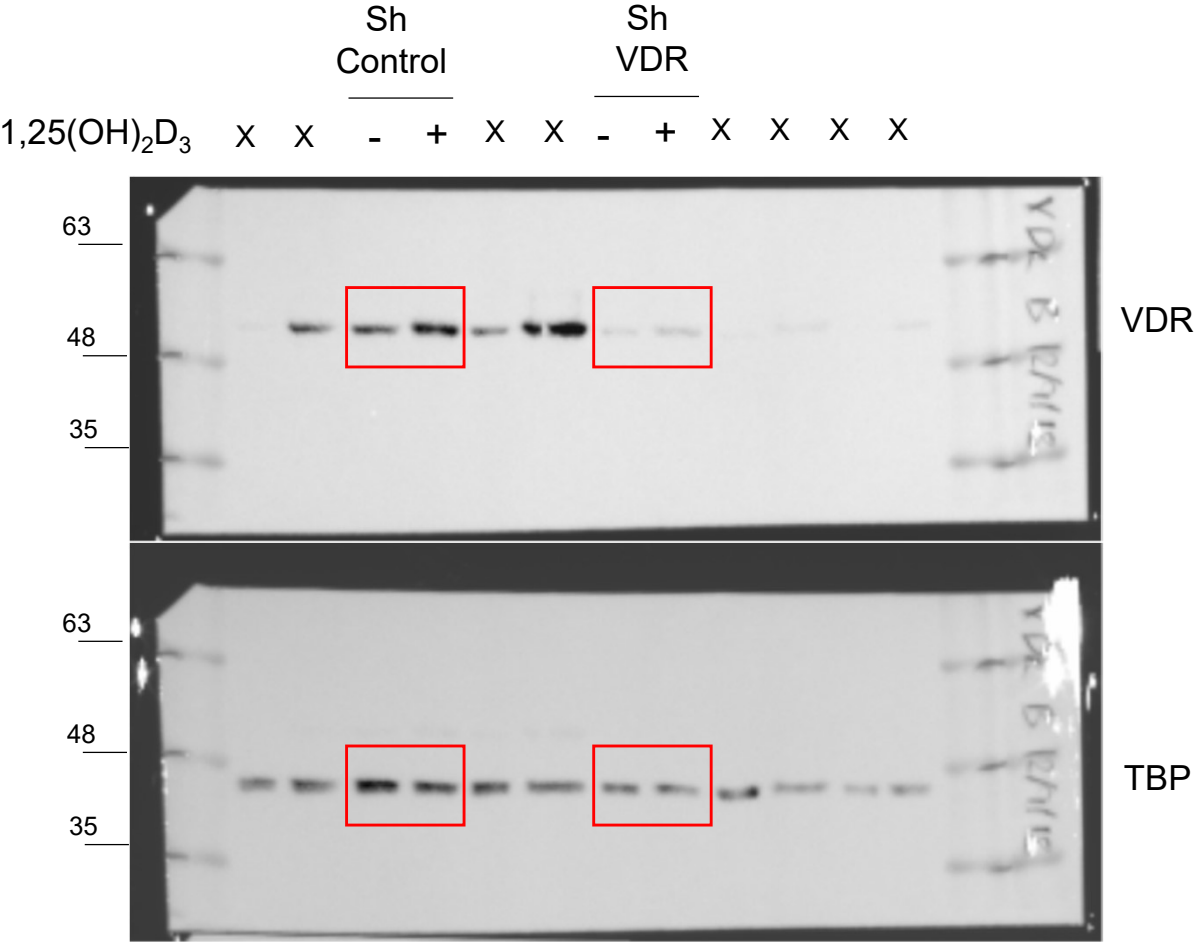

Supplement: Figure 1—source data 1. [file elife-86913-fig1-data1.zip › Fig1_source data/Fig 1G_raw TIFFand PDF/PDF Fig 1G.pdf]

Figure 1H

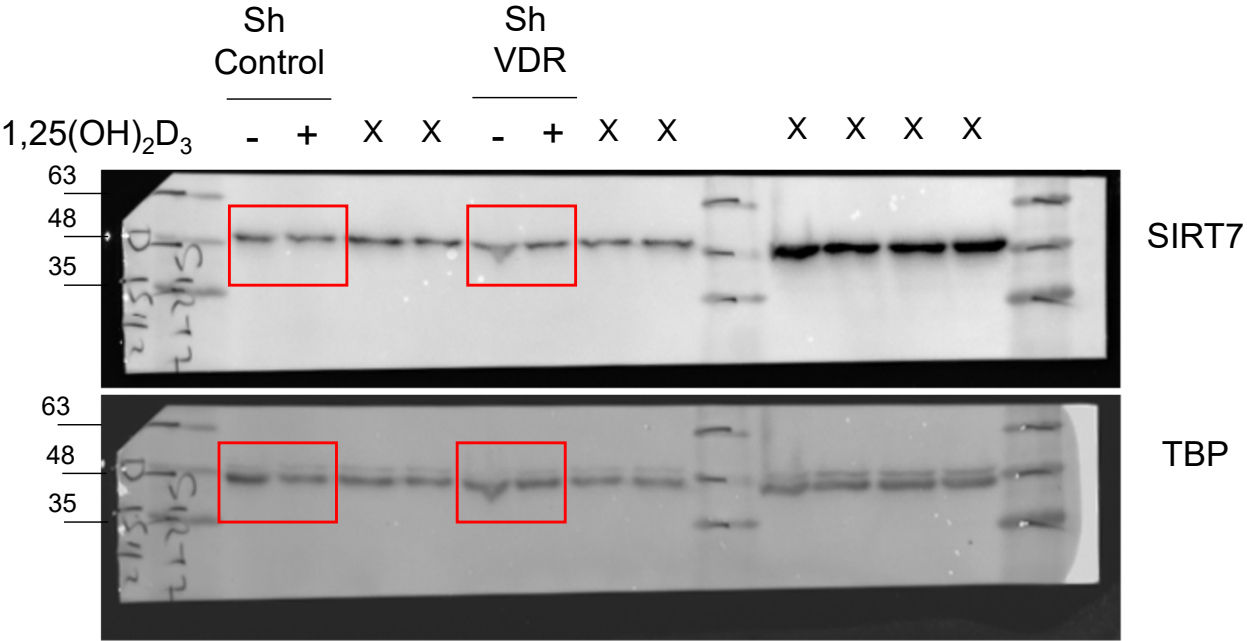

Supplement: Figure 1—source data 1. [file elife-86913-fig1-data1.zip › Fig1_source data/Fig 1H_raw DATA_PDF and TIFF/Fig 1H_raw PDF.pdf]

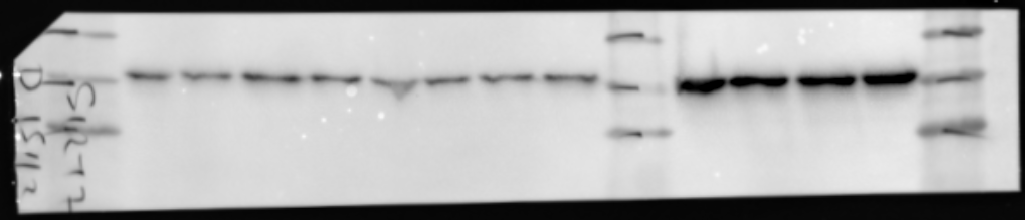

Supplement: Figure 1—source data 1. [file elife-86913-fig1-data1.zip › Fig1_source data/Fig 1H_raw DATA_PDF and TIFF/Figure 1H_scan_SIRT7.tif]

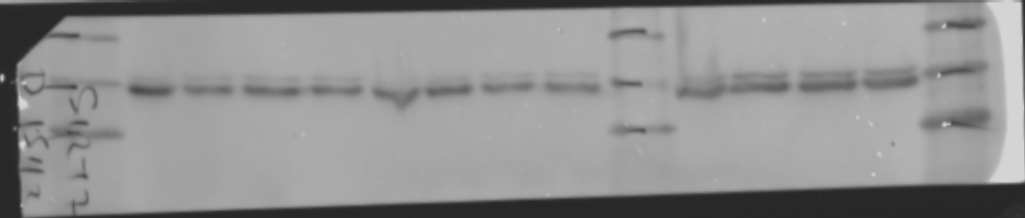

Supplement: Figure 1—source data 1. [file elife-86913-fig1-data1.zip › Fig1_source data/Fig 1H_raw DATA_PDF and TIFF/Figure 1H_scan_TBP.tif]

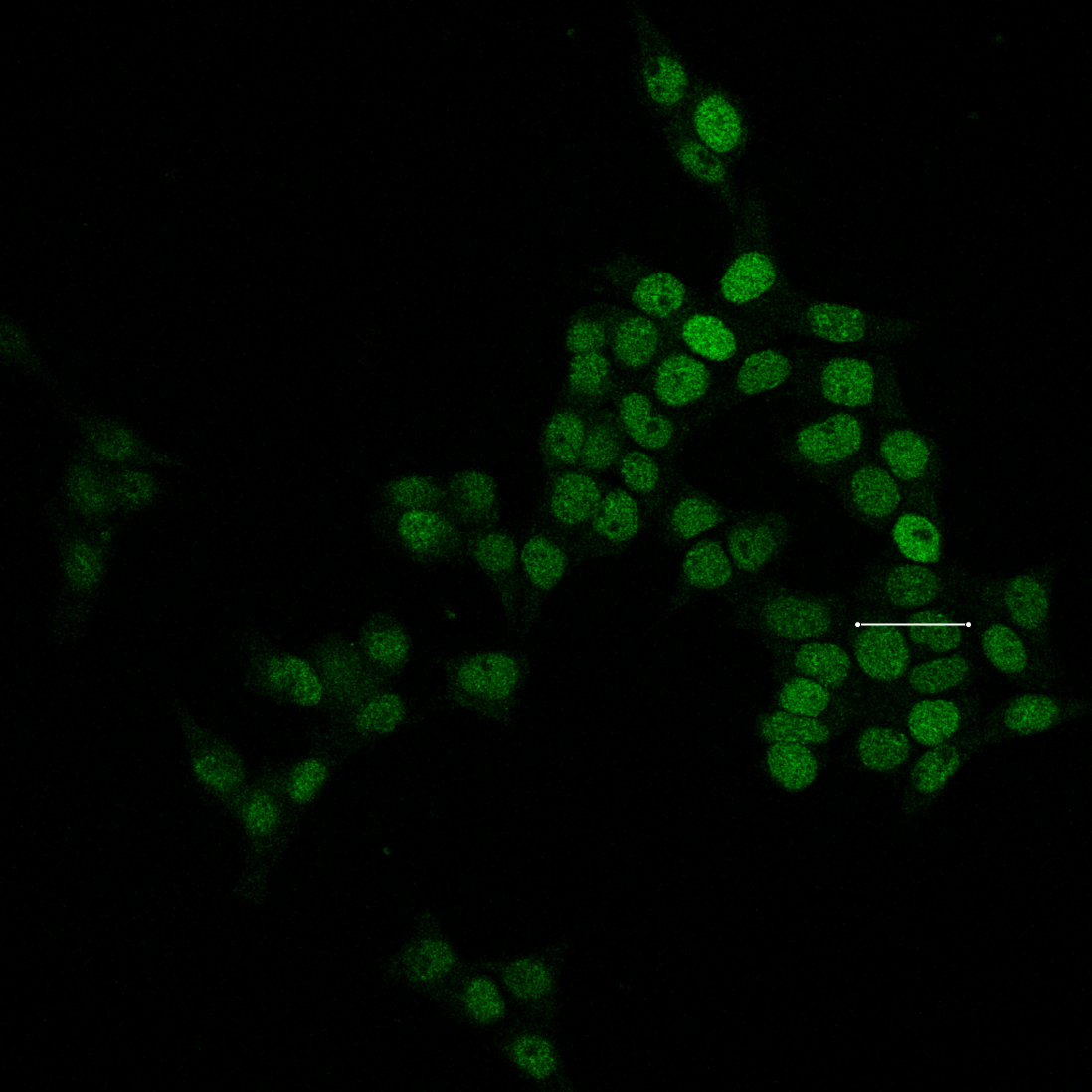

Supplement: Figure 1—source data 1. [file elife-86913-fig1-data1.zip › Fig1_source data/Fig. 1F_raw TIFF/Sh Cnt.tif]

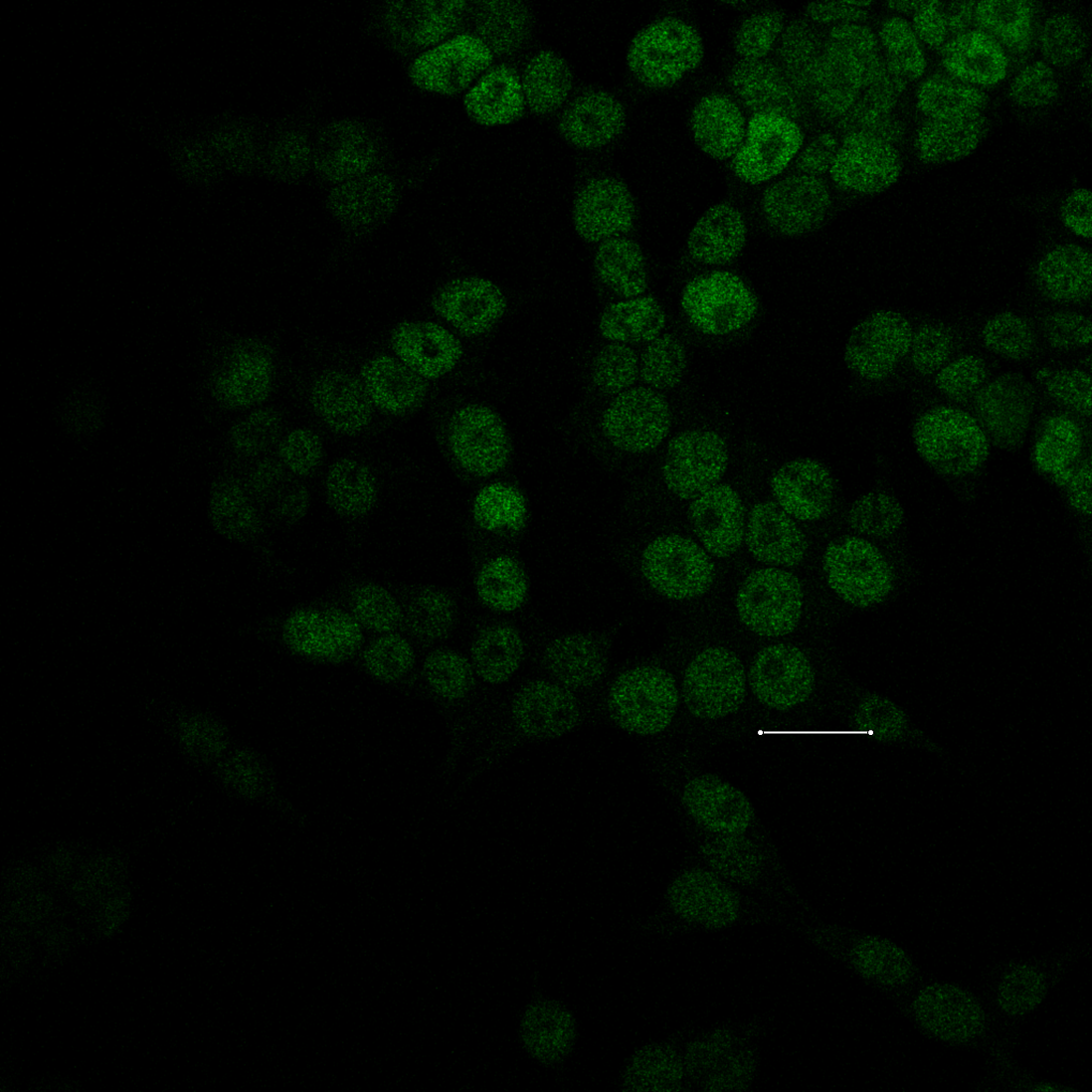

Supplement: Figure 1—source data 1. [file elife-86913-fig1-data1.zip › Fig1_source data/Fig. 1F_raw TIFF/Sh VDR.tif]

Figure 2C

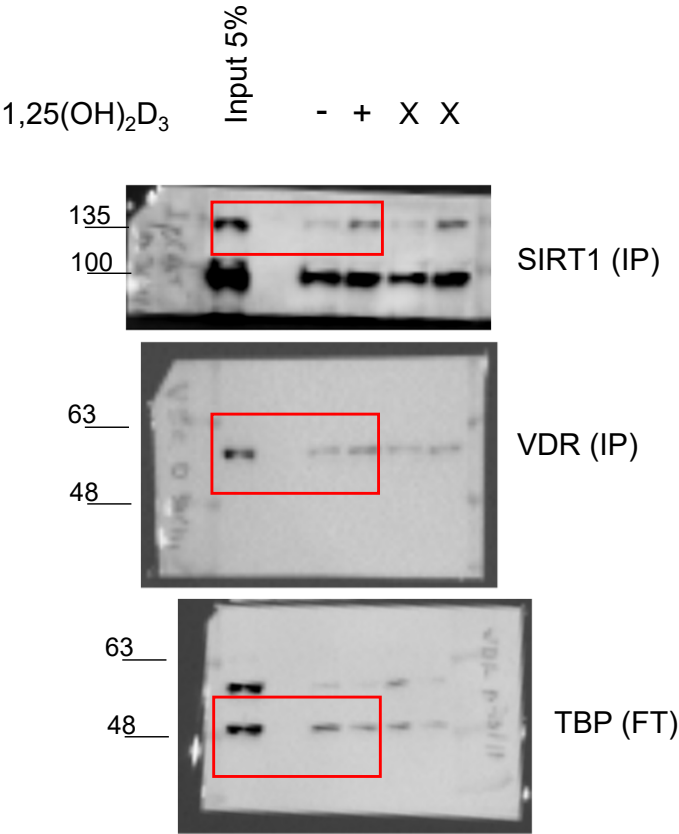

Supplement: Figure 2—source data 1. [file elife-86913-fig2-data1.zip › Fig2_source data/Fig 2C_raw TIFFand PDF/Fig.2C_raw data_pdf.pdf]

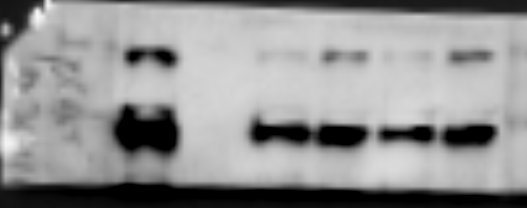

Supplement: Figure 2—source data 1. [file elife-86913-fig2-data1.zip › Fig2_source data/Fig 2C_raw TIFFand PDF/Figue 2C_scan_SIRT1.tif]

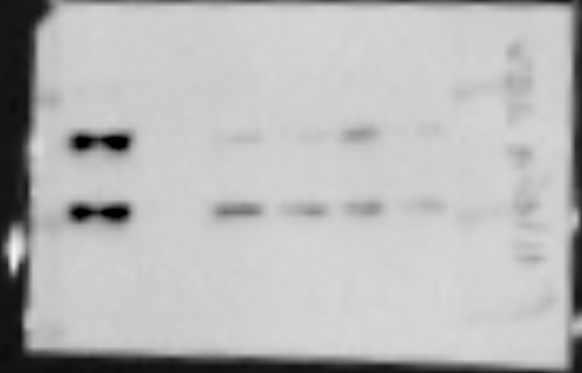

Supplement: Figure 2—source data 1. [file elife-86913-fig2-data1.zip › Fig2_source data/Fig 2C_raw TIFFand PDF/Figue 2C_scan_tbp.tif]

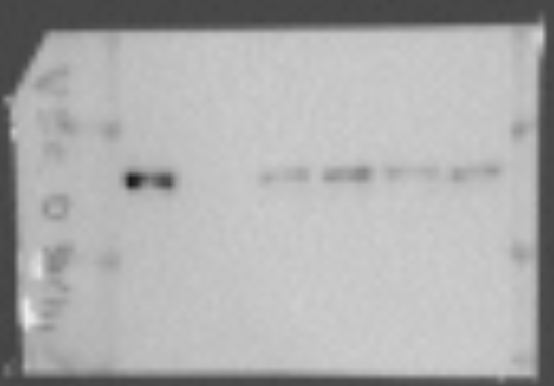

Supplement: Figure 2—source data 1. [file elife-86913-fig2-data1.zip › Fig2_source data/Fig 2C_raw TIFFand PDF/Figue 2C_scan_VDR.tif]

Figure 2D

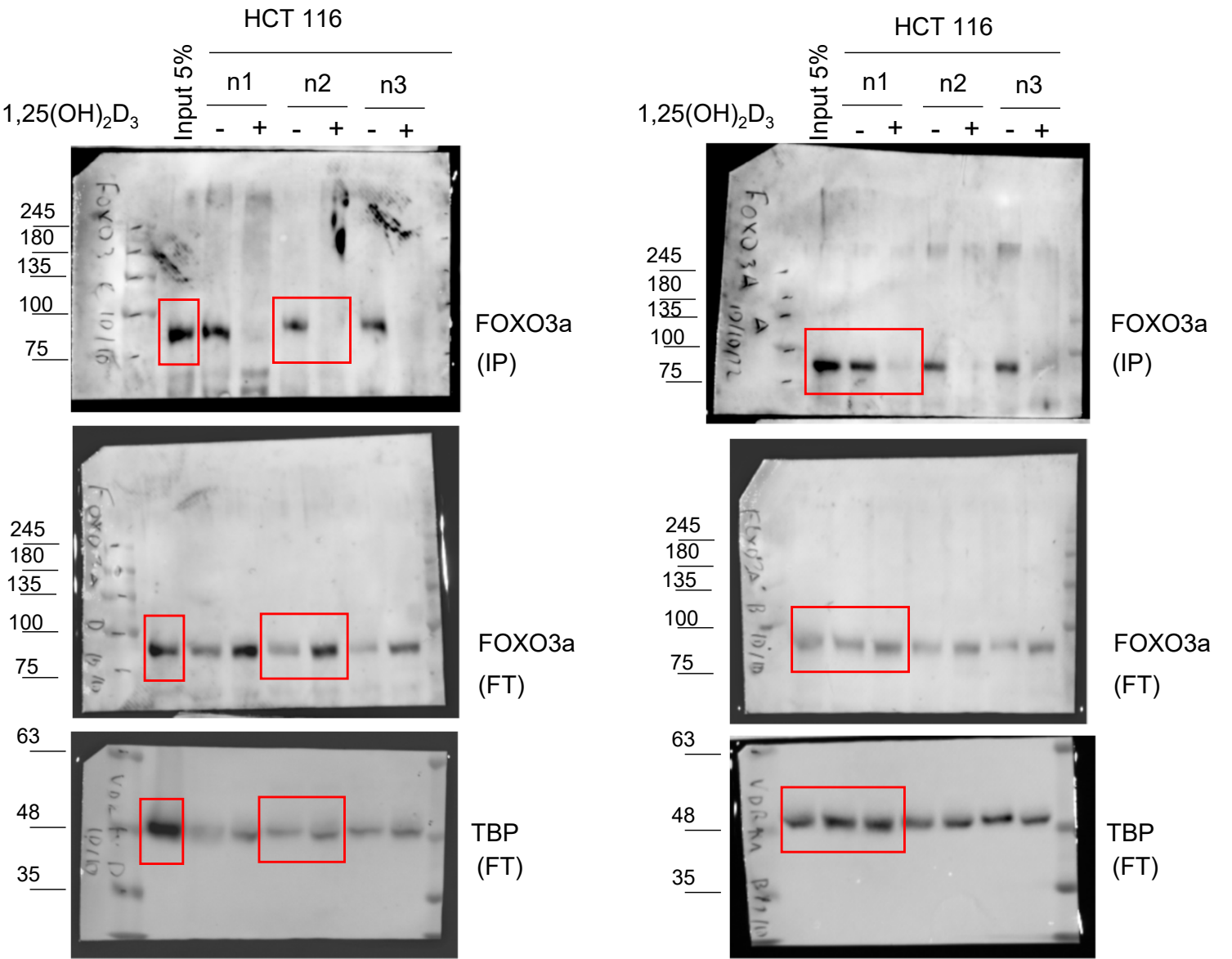

Supplement: Figure 2—source data 1. [file elife-86913-fig2-data1.zip › Fig2_source data/Fig 2D_raw TIFFand PDF/Fig.2D_raw data_pdf.pdf]

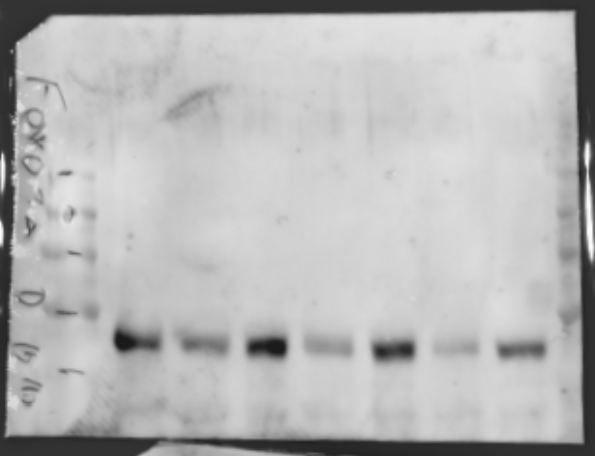

Supplement: Figure 2—source data 1. [file elife-86913-fig2-data1.zip › Fig2_source data/Fig 2D_raw TIFFand PDF/Figure 2D_scan_HCT116_FOXO3a_FT.tif]

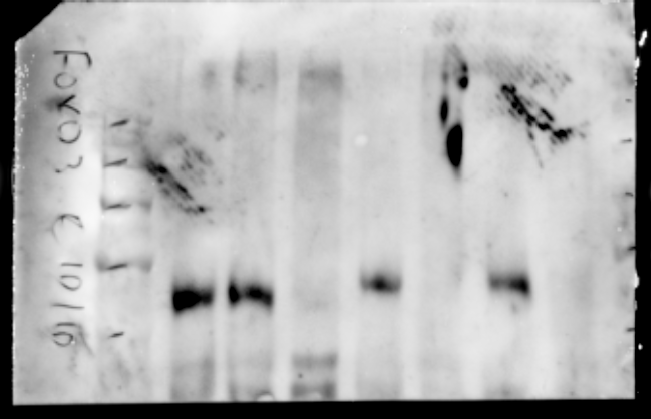

Supplement: Figure 2—source data 1. [file elife-86913-fig2-data1.zip › Fig2_source data/Fig 2D_raw TIFFand PDF/Figure 2D_scan_HCT116_FOXO3a_IP.tif]

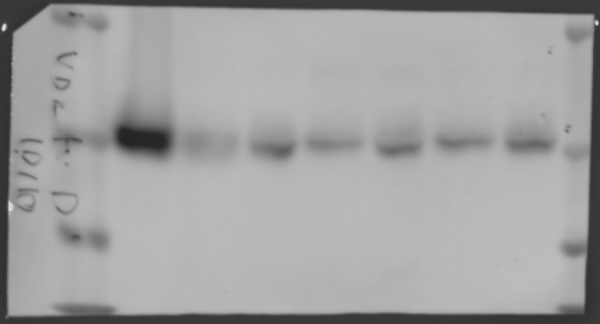

Supplement: Figure 2—source data 1. [file elife-86913-fig2-data1.zip › Fig2_source data/Fig 2D_raw TIFFand PDF/Figure 2D_scan_HCT116_TBP_FT.tif]

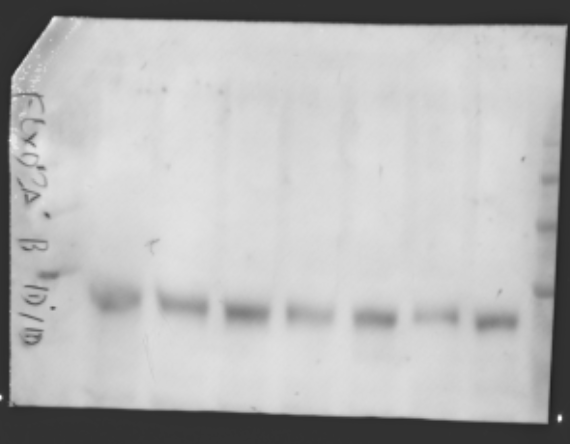

Supplement: Figure 2—source data 1. [file elife-86913-fig2-data1.zip › Fig2_source data/Fig 2D_raw TIFFand PDF/Figure 2D_scan_HT29_FOXO3a_FT.tif]

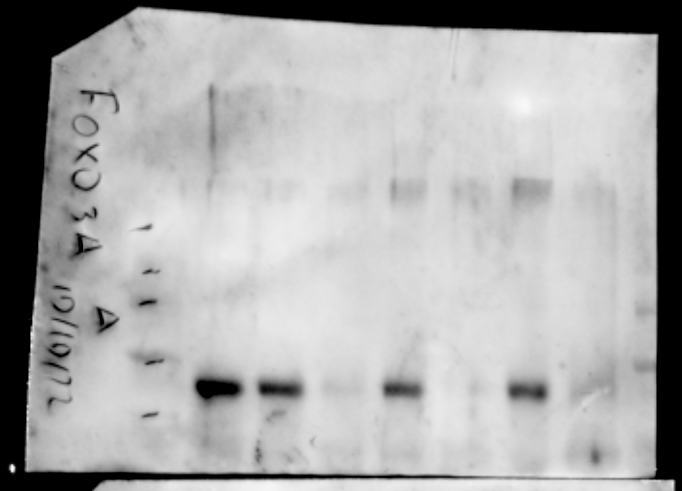

Supplement: Figure 2—source data 1. [file elife-86913-fig2-data1.zip › Fig2_source data/Fig 2D_raw TIFFand PDF/Figure 2D_scan_HT29_FOXO3a_IP.tif]

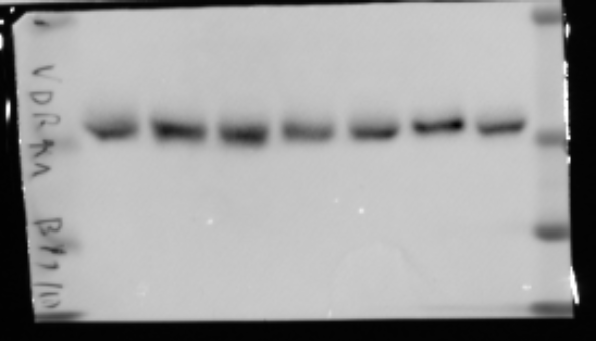

Supplement: Figure 2—source data 1. [file elife-86913-fig2-data1.zip › Fig2_source data/Fig 2D_raw TIFFand PDF/Figure 2D_scan_HT29_TBP_FT.tif]

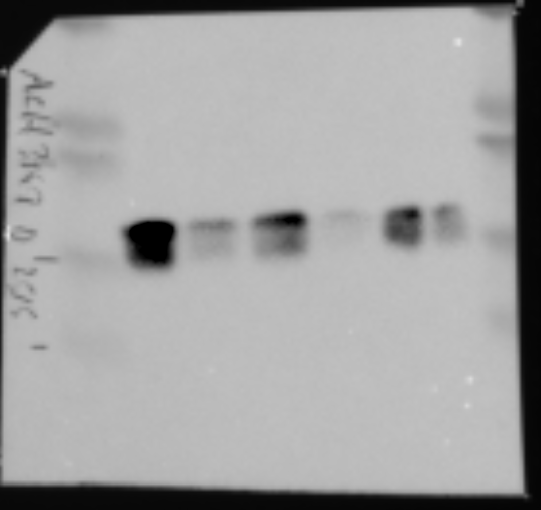

Supplement: Figure 2—source data 1. [file elife-86913-fig2-data1.zip › Fig2_source data/Fig 2E_raw TIFFand PDF/Figue 2E_scan_HCT116_AceH3K9.tif]

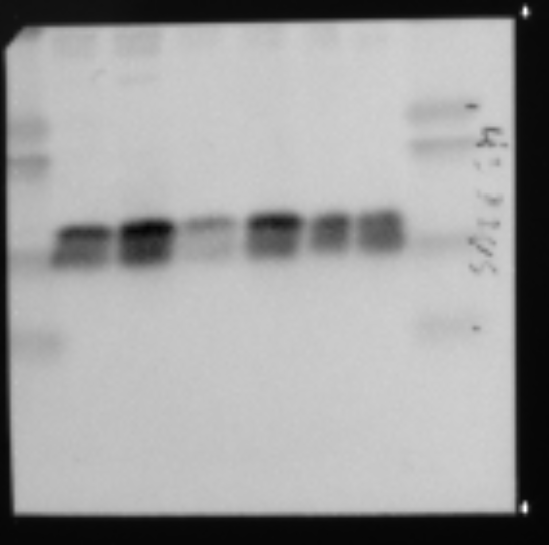

Supplement: Figure 2—source data 1. [file elife-86913-fig2-data1.zip › Fig2_source data/Fig 2E_raw TIFFand PDF/Figue 2E_scan_HCT116_H3.tif]

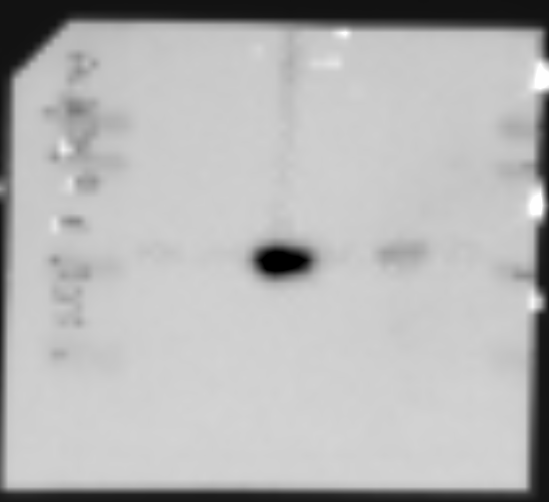

Supplement: Figure 2—source data 1. [file elife-86913-fig2-data1.zip › Fig2_source data/Fig 2E_raw TIFFand PDF/Figue 2E_scan_HT29_AceH3K9.tif]

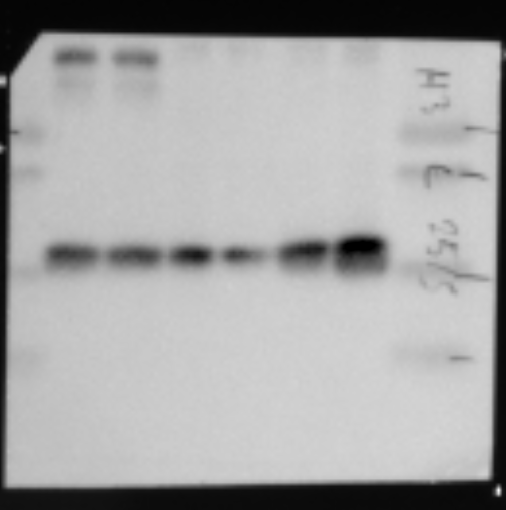

Supplement: Figure 2—source data 1. [file elife-86913-fig2-data1.zip › Fig2_source data/Fig 2E_raw TIFFand PDF/Figue 2E_scan_HT29_H3.tif]

Figure 2E

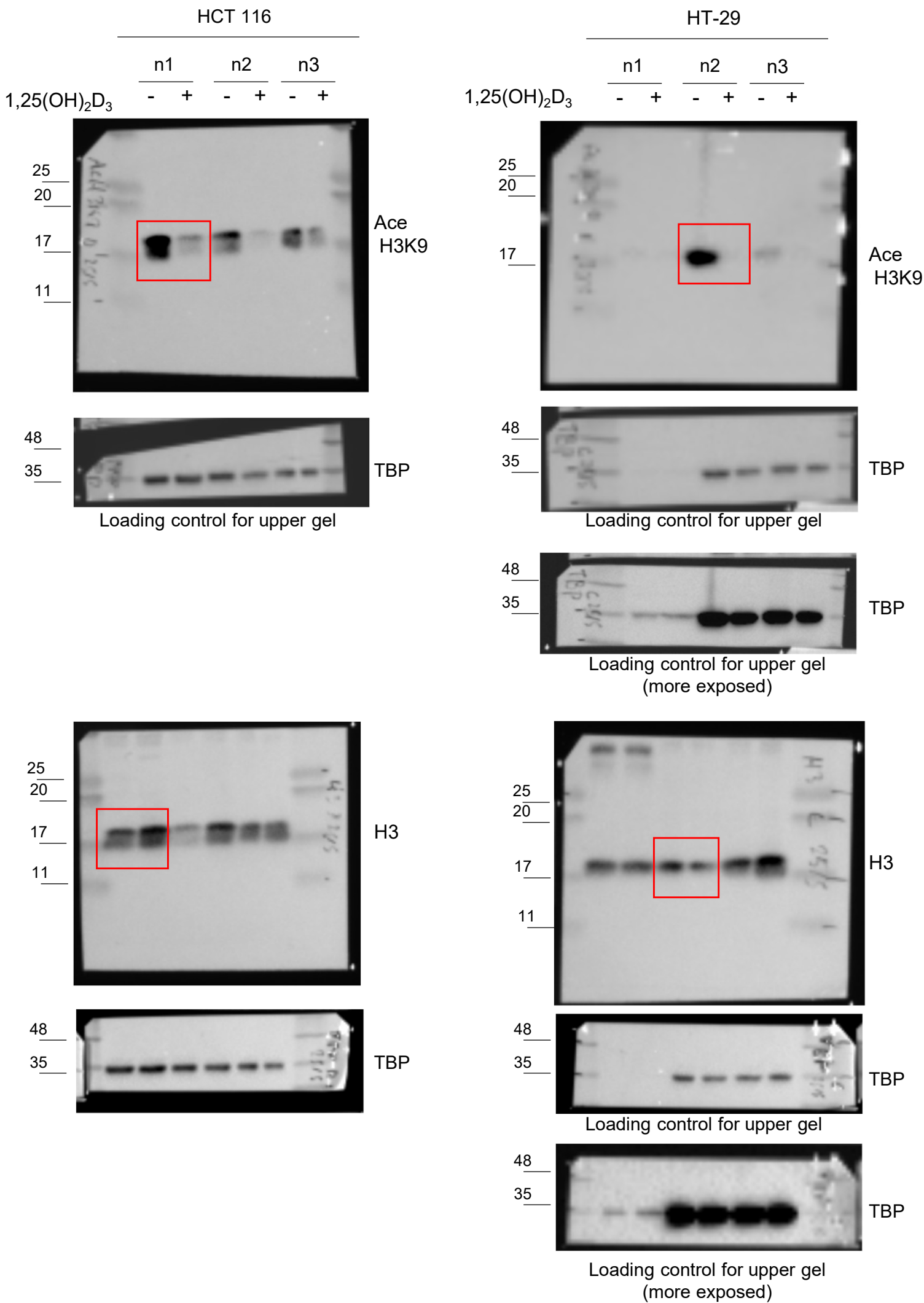

Supplement: Figure 2—source data 1. [file elife-86913-fig2-data1.zip › Fig2_source data/Fig 2E_raw TIFFand PDF/PDF Fig 2E.pdf]

Figure 2F

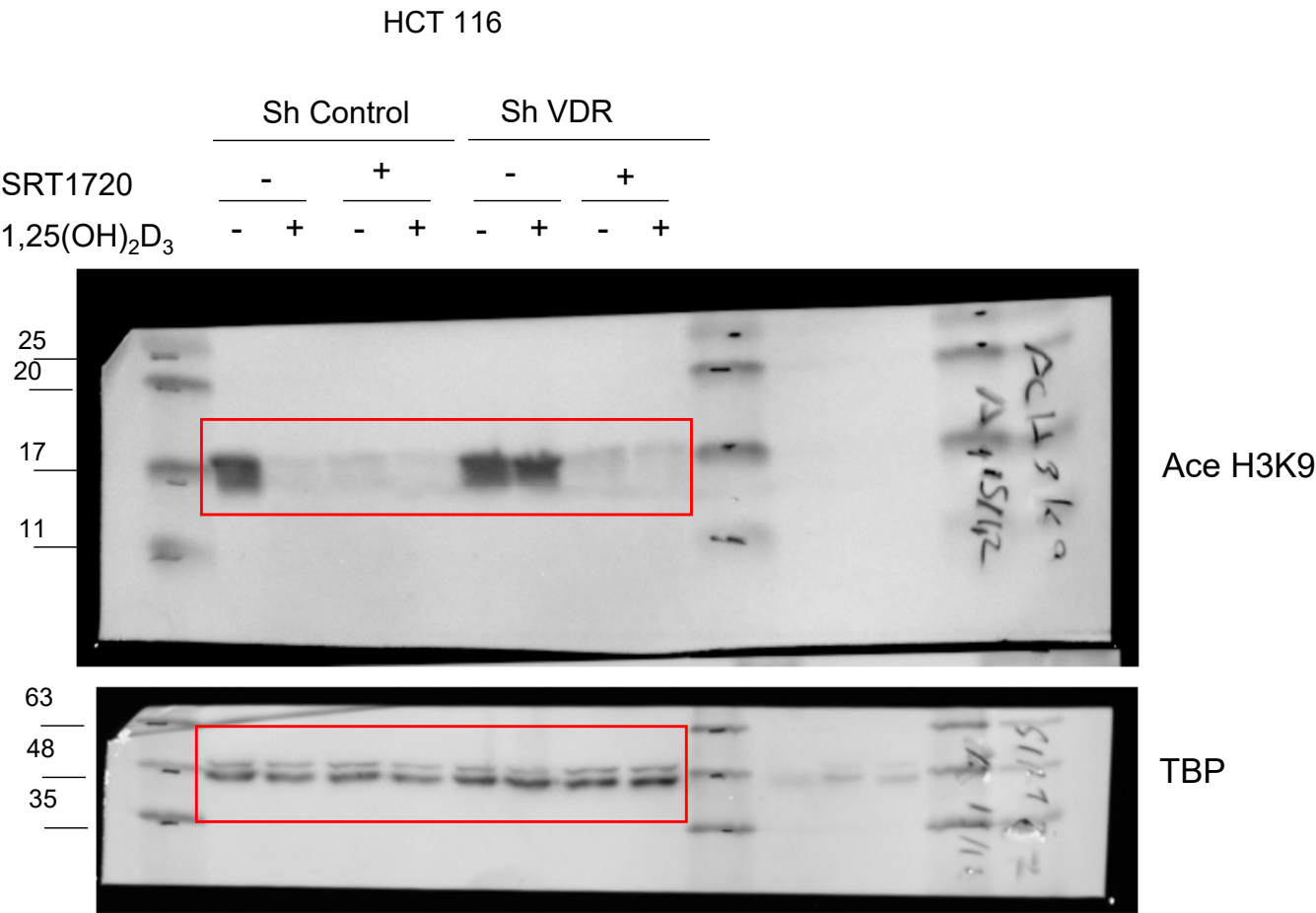

Supplement: Figure 2—source data 1. [file elife-86913-fig2-data1.zip › Fig2_source data/Fig 2F_raw TIFFand PDF/Fig.2F_raw data_pdf.pdf]

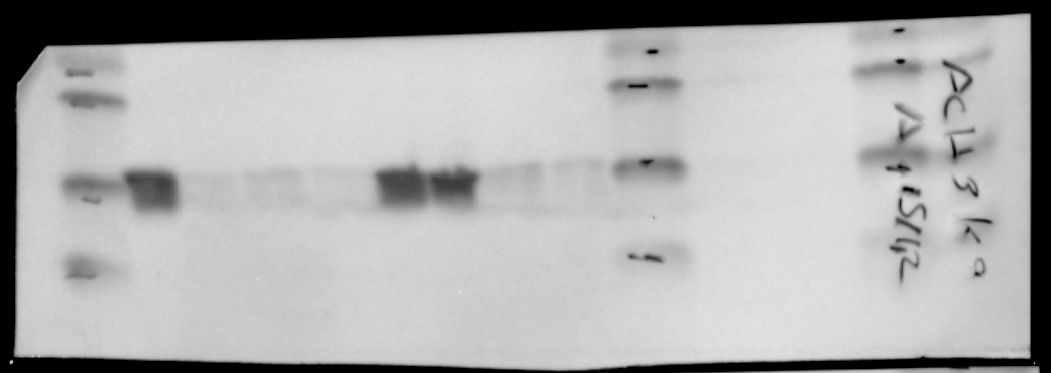

Supplement: Figure 2—source data 1. [file elife-86913-fig2-data1.zip › Fig2_source data/Fig 2F_raw TIFFand PDF/Figure 2F_scan_AceH3K9.tif]

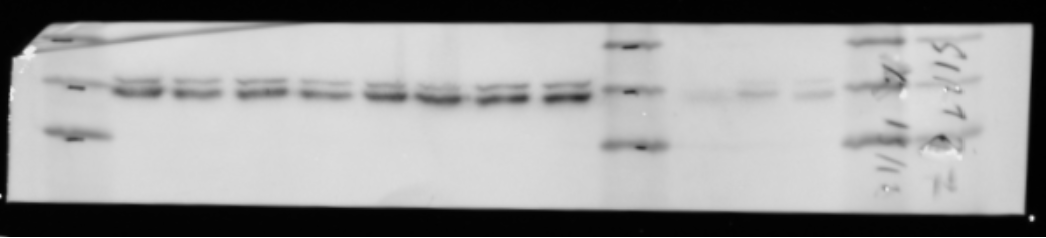

Supplement: Figure 2—source data 1. [file elife-86913-fig2-data1.zip › Fig2_source data/Fig 2F_raw TIFFand PDF/Figure 2F_scan_YBP.tif]

Figure 2G

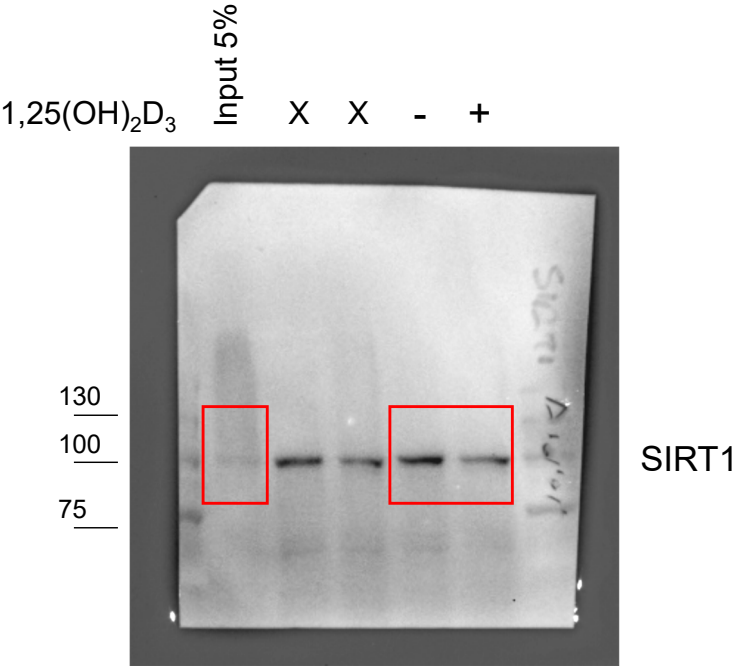

Supplement: Figure 2—source data 1. [file elife-86913-fig2-data1.zip › Fig2_source data/Fig 2G_raw TIFFand PDF/Fig.2G_raw data_pdf.pdf]

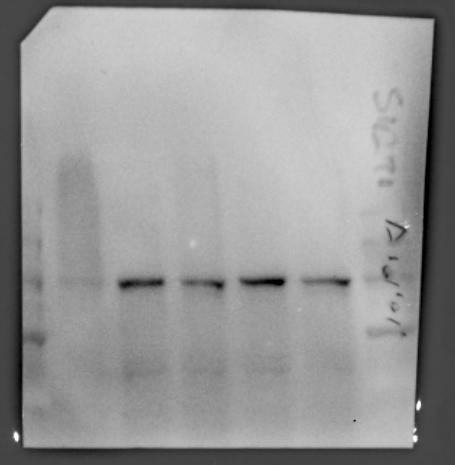

Supplement: Figure 2—source data 1. [file elife-86913-fig2-data1.zip › Fig2_source data/Fig 2G_raw TIFFand PDF/Figure 2G_scan_SIRT1.tif]

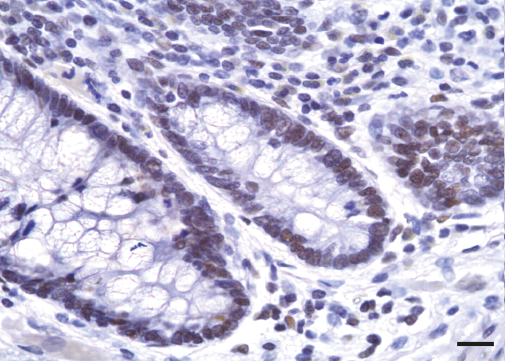

Supplement: Figure 3—source data 1. [file elife-86913-fig3-data1.zip › Fig3_source data/Fig.3D_ TIFF/SIRT1 Non-Tumor.tif]

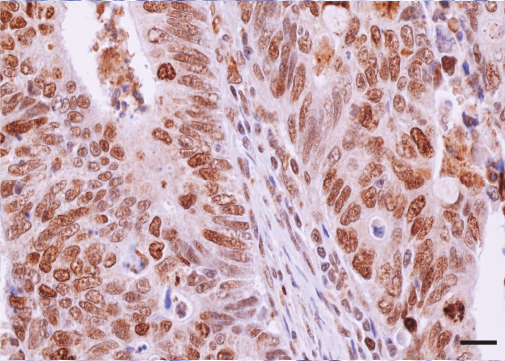

Supplement: Figure 3—source data 1. [file elife-86913-fig3-data1.zip › Fig3_source data/Fig.3D_ TIFF/SIRT1 Tumor.tif]

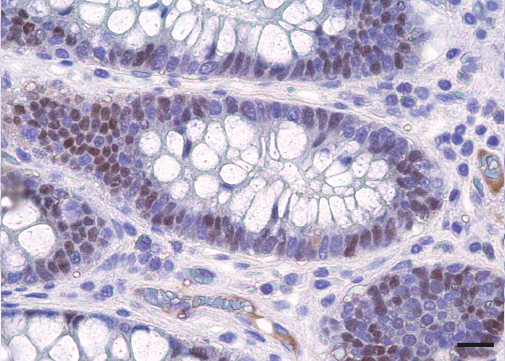

Supplement: Figure 3—source data 1. [file elife-86913-fig3-data1.zip › Fig3_source data/Fig.3D_ TIFF/VDR Non-Tumor.tif]

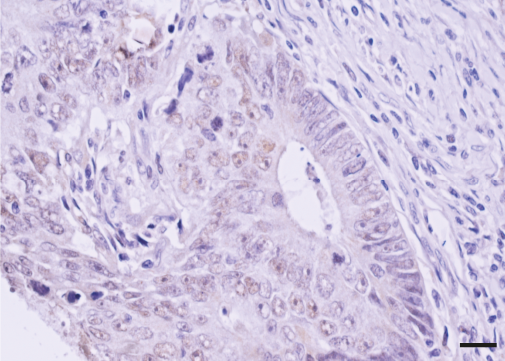

Supplement: Figure 3—source data 1. [file elife-86913-fig3-data1.zip › Fig3_source data/Fig.3D_ TIFF/VDR Tumor.tif]

Figure 3F

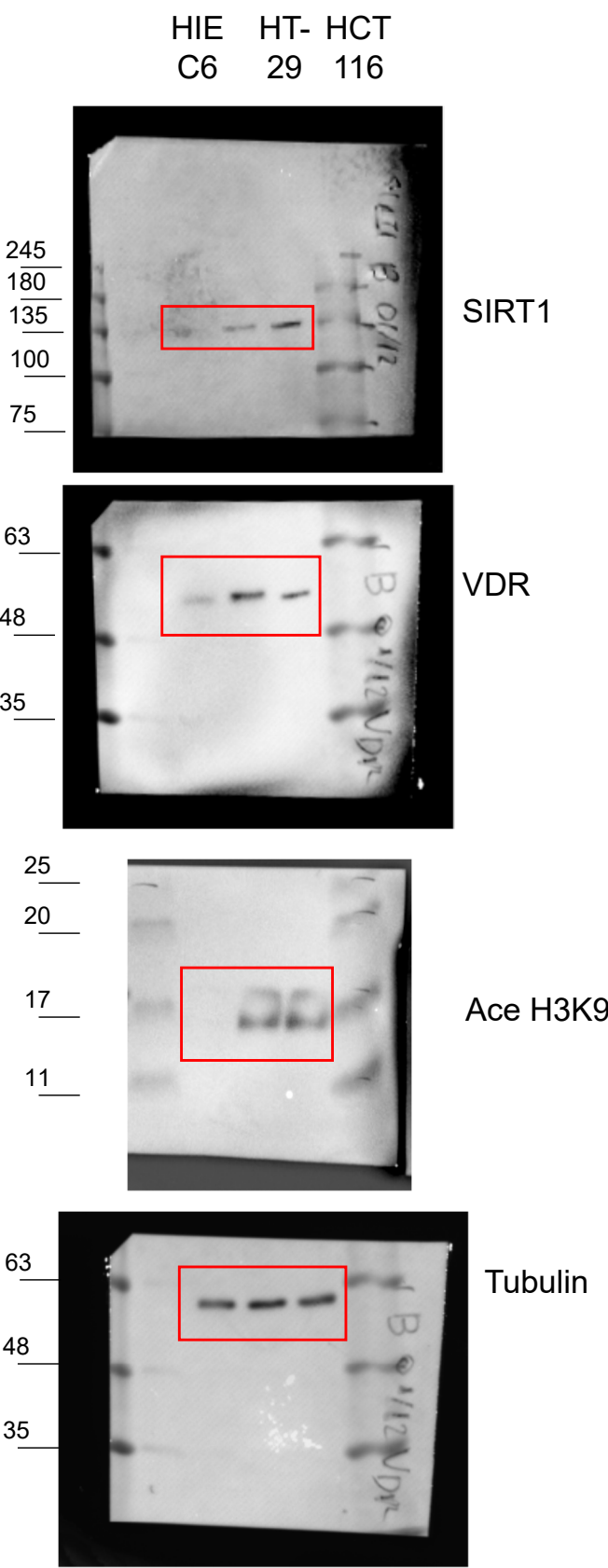

Supplement: Figure 3—source data 1. [file elife-86913-fig3-data1.zip › Fig3_source data/Fig.3F_raw data_pdf and TIFF/Fig.3F_raw data_pdf.pdf]

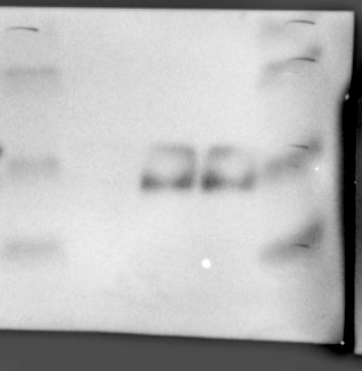

Supplement: Figure 3—source data 1. [file elife-86913-fig3-data1.zip › Fig3_source data/Fig.3F_raw data_pdf and TIFF/Figure 3F_scan_AceH3K9.tif]

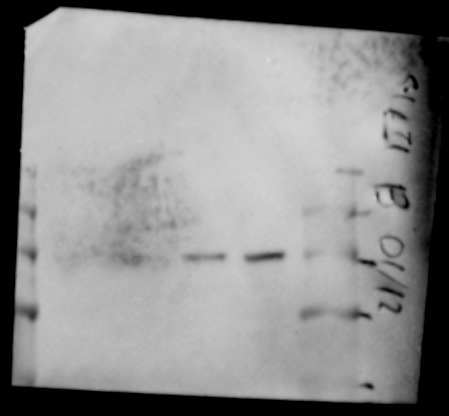

Supplement: Figure 3—source data 1. [file elife-86913-fig3-data1.zip › Fig3_source data/Fig.3F_raw data_pdf and TIFF/Figure 3F_scan_SIRT1.tif]

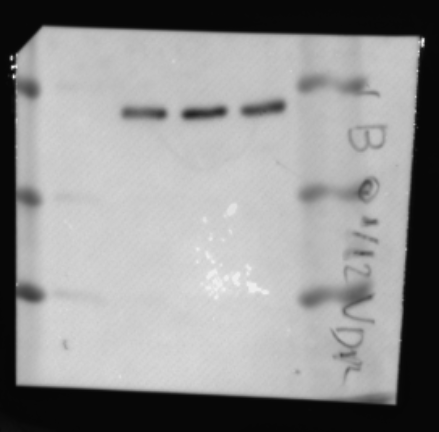

Supplement: Figure 3—source data 1. [file elife-86913-fig3-data1.zip › Fig3_source data/Fig.3F_raw data_pdf and TIFF/Figure 3F_scan_tubulin.tif]

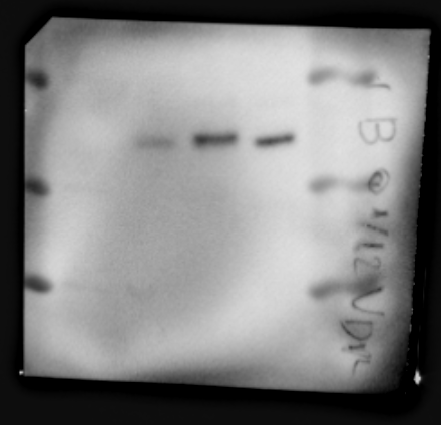

Supplement: Figure 3—source data 1. [file elife-86913-fig3-data1.zip › Fig3_source data/Fig.3F_raw data_pdf and TIFF/Figure 3F_scan_vdr.tif]

Figure 3G

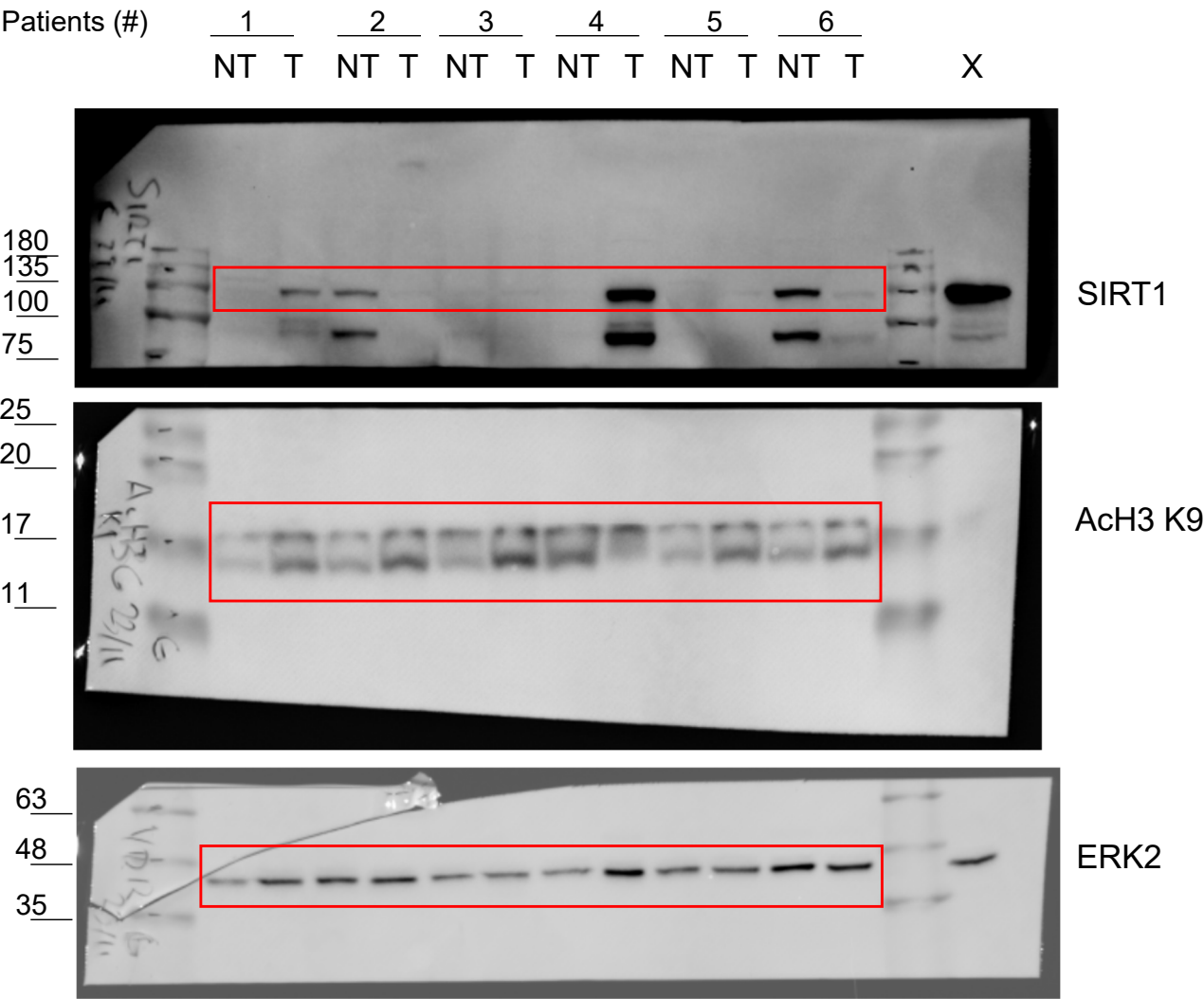

Supplement: Figure 3—source data 1. [file elife-86913-fig3-data1.zip › Fig3_source data/Fig.3G_raw data_pdf and TIFF/Fig.3G_raw data_pdf.pdf]

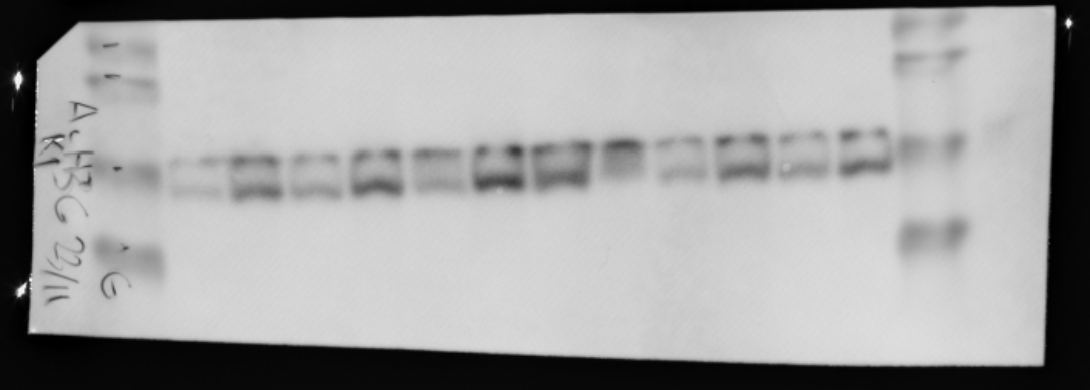

Supplement: Figure 3—source data 1. [file elife-86913-fig3-data1.zip › Fig3_source data/Fig.3G_raw data_pdf and TIFF/Figure 3G_scan_AceH3K9.tif]

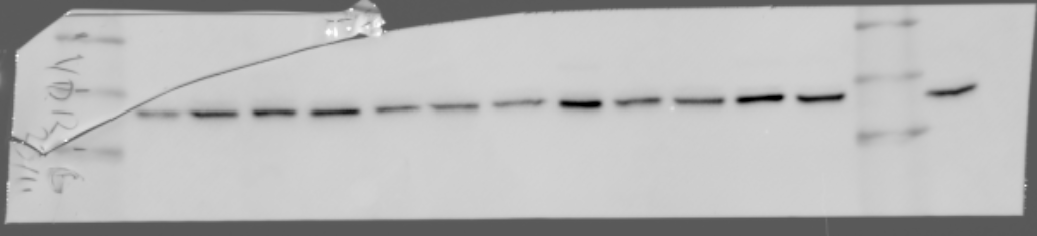

Supplement: Figure 3—source data 1. [file elife-86913-fig3-data1.zip › Fig3_source data/Fig.3G_raw data_pdf and TIFF/Figure 3G_scan_ERK2.tif]

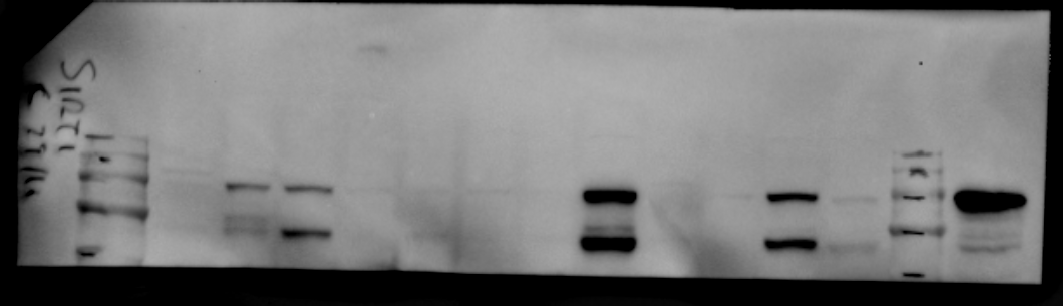

Supplement: Figure 3—source data 1. [file elife-86913-fig3-data1.zip › Fig3_source data/Fig.3G_raw data_pdf and TIFF/Figure 3G_scan_SIRT1.tif]

Figure 3H

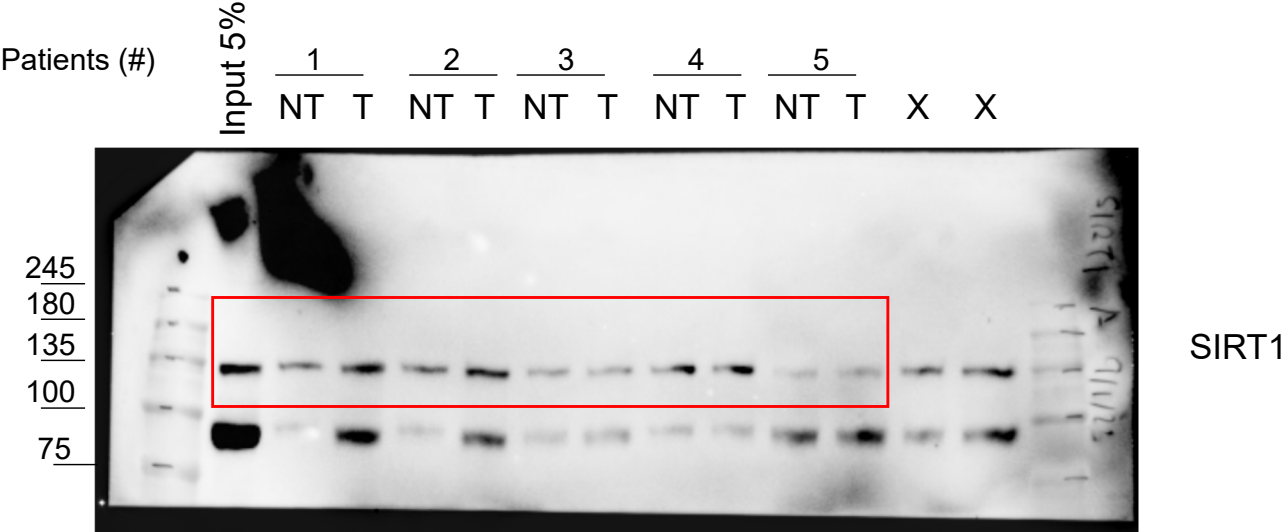

Supplement: Figure 3—source data 1. [file elife-86913-fig3-data1.zip › Fig3_source data/Fig.3H_raw data_pdf and TIFF/Fig.3H_raw data_pdf.pdf]

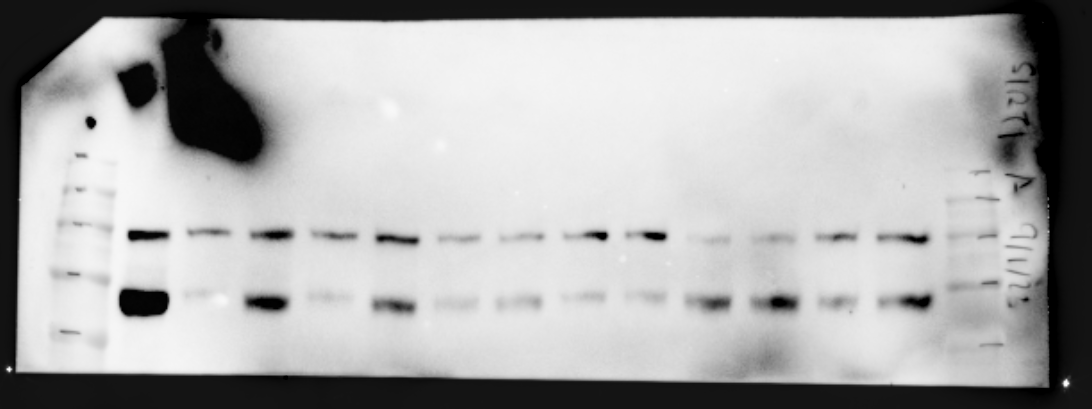

Supplement: Figure 3—source data 1. [file elife-86913-fig3-data1.zip › Fig3_source data/Fig.3H_raw data_pdf and TIFF/Figure 3H_scan_SIRT1.tif]

Figure 3I

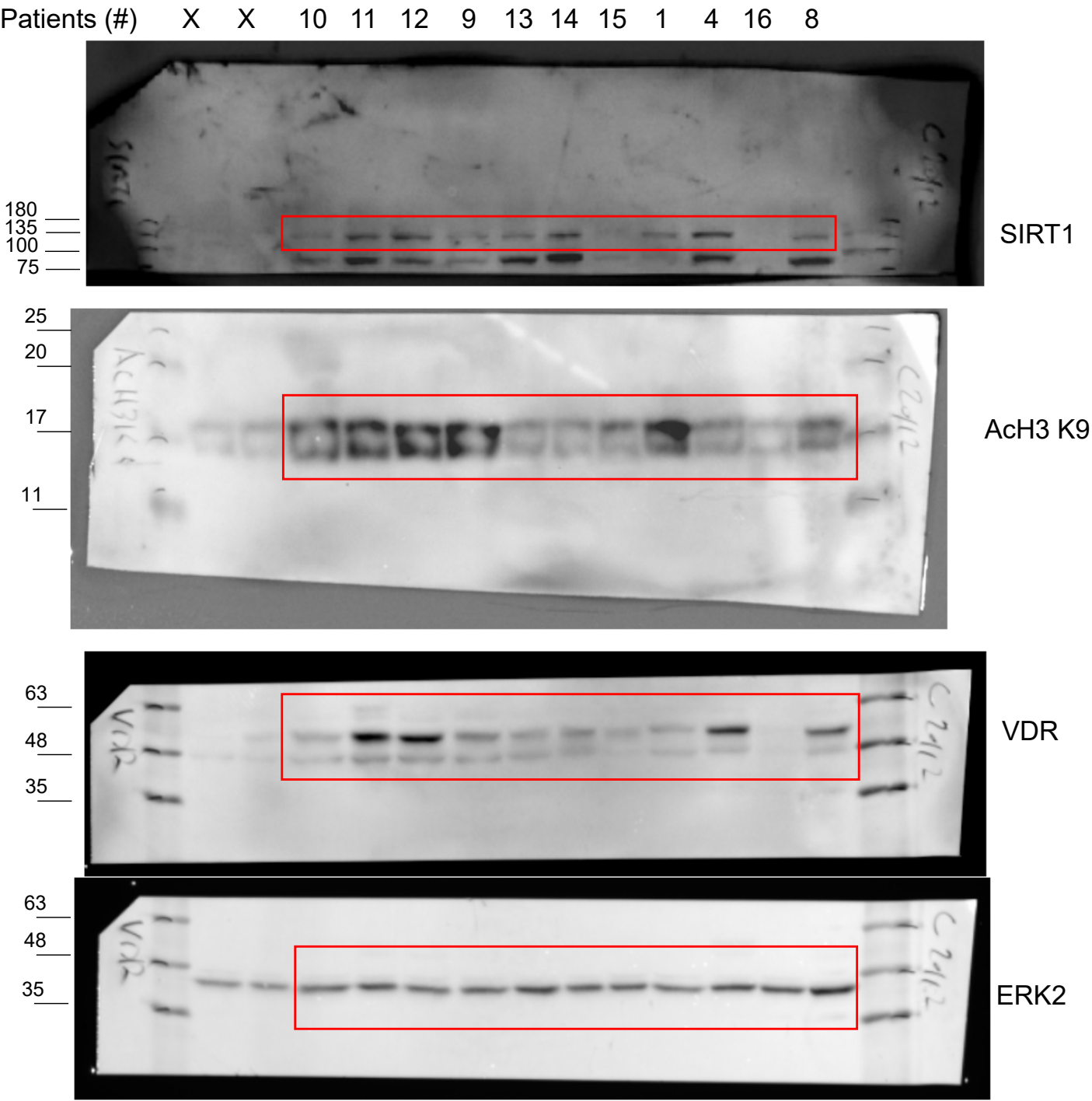

Supplement: Figure 3—source data 1. [file elife-86913-fig3-data1.zip › Fig3_source data/Fig.3I_raw data_pdf and TIFF/Fig.3I_raw data_pdf.pdf]

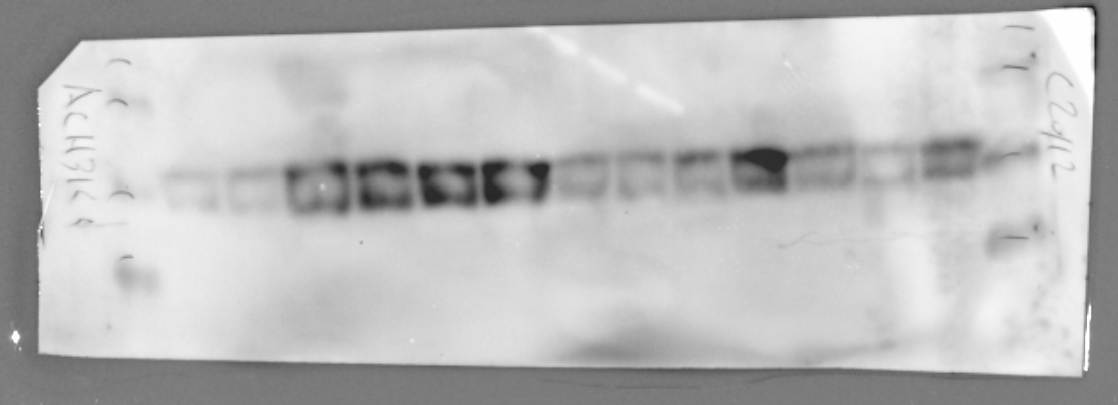

Supplement: Figure 3—source data 1. [file elife-86913-fig3-data1.zip › Fig3_source data/Fig.3I_raw data_pdf and TIFF/Figure 3I_scan_AceH3K9.tif]

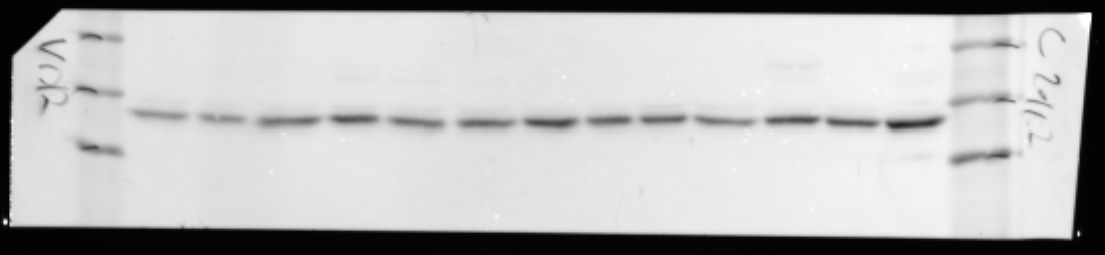

Supplement: Figure 3—source data 1. [file elife-86913-fig3-data1.zip › Fig3_source data/Fig.3I_raw data_pdf and TIFF/Figure 3I_scan_ERK2.tif]

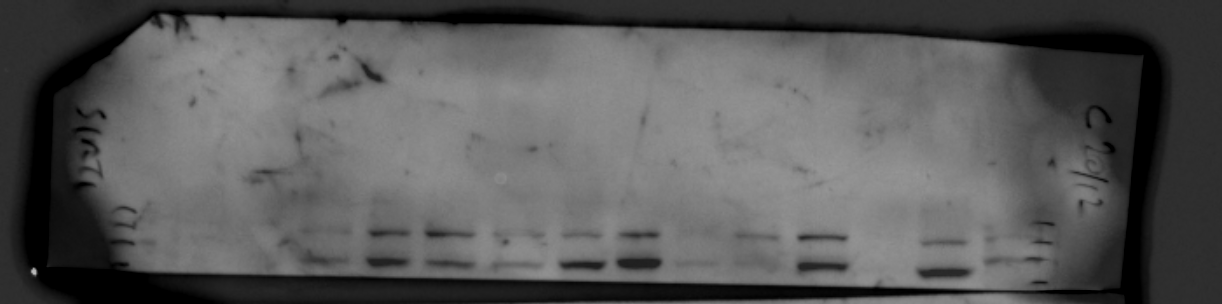

Supplement: Figure 3—source data 1. [file elife-86913-fig3-data1.zip › Fig3_source data/Fig.3I_raw data_pdf and TIFF/Figure 3I_scan_SIRT1.tif]

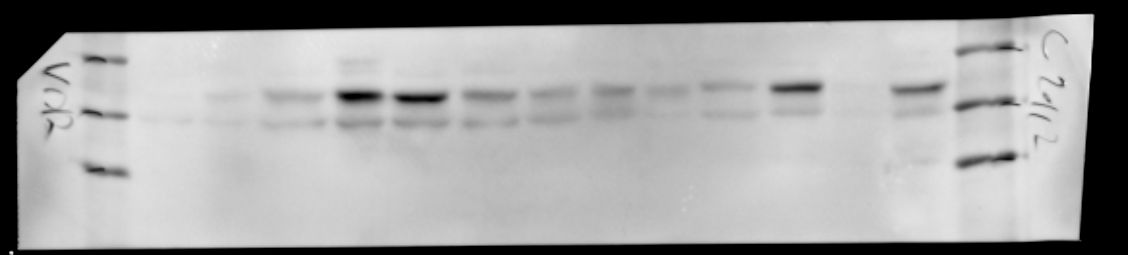

Supplement: Figure 3—source data 1. [file elife-86913-fig3-data1.zip › Fig3_source data/Fig.3I_raw data_pdf and TIFF/Figure 3I_scan_VDR.tif]

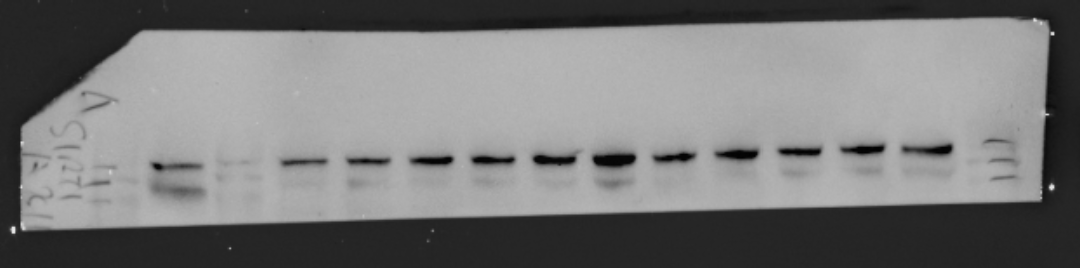

Supplement: Figure 3—source data 1. [file elife-86913-fig3-data1.zip › Fig3_source data/Fig.3I_raw data_pdf and TIFF/Figure 3J_scan_SIRT1.tif]

Figure 3J

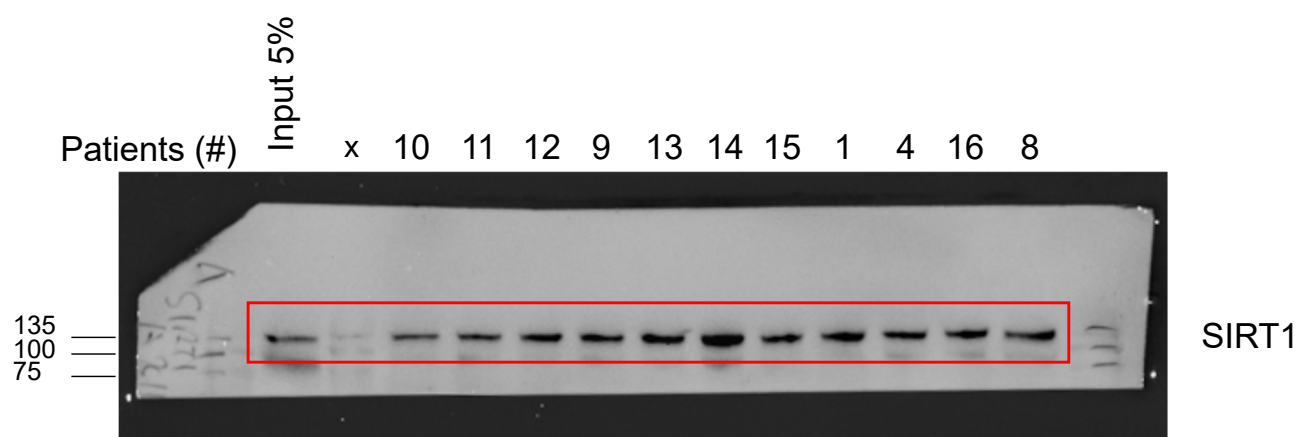

Supplement: Figure 3—source data 1. [file elife-86913-fig3-data1.zip › Fig3_source data/Fig.3J_raw data_pdf and TIFF/Fig.3J_raw data_pdf.pdf]

Figure 4A

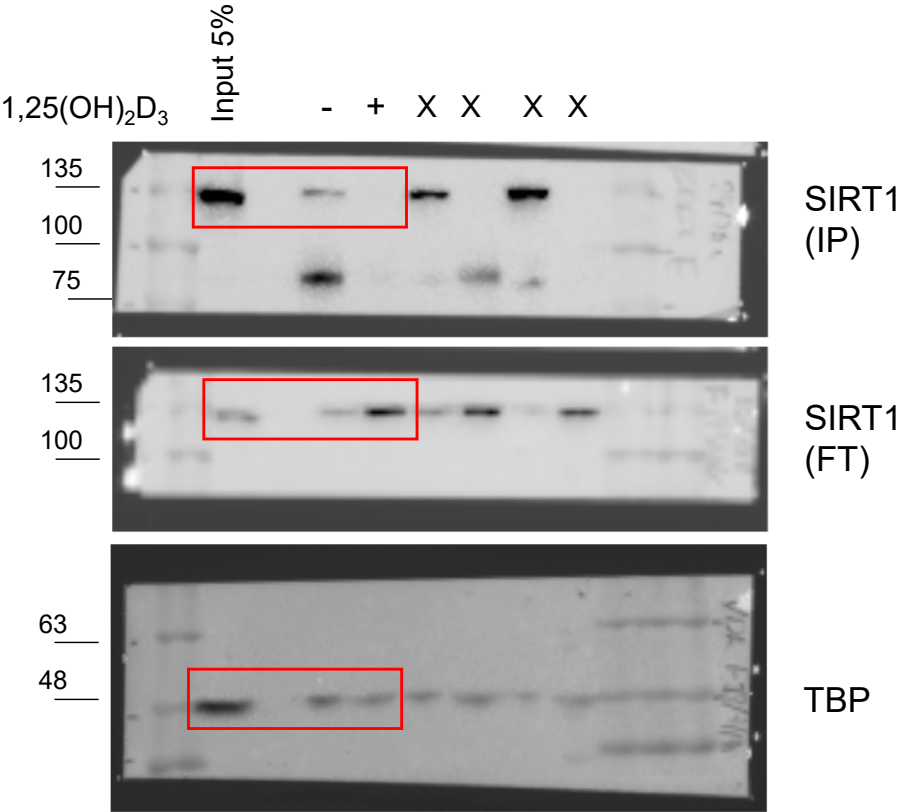

Supplement: Figure 4—source data 1. [file elife-86913-fig4-data1.zip › Fig4_source data/Fig.4A_raw data_pdf and TIFF/Fig.4A_raw data_pdf.pdf]

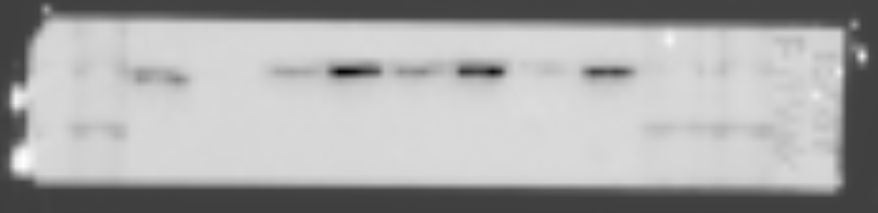

Supplement: Figure 4—source data 1. [file elife-86913-fig4-data1.zip › Fig4_source data/Fig.4A_raw data_pdf and TIFF/Figure 4A_scan_SIRT1 FT.tif]

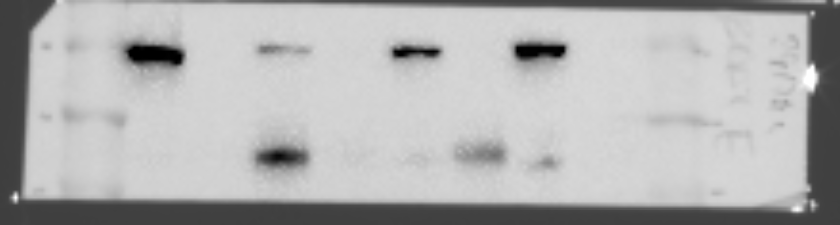

Supplement: Figure 4—source data 1. [file elife-86913-fig4-data1.zip › Fig4_source data/Fig.4A_raw data_pdf and TIFF/Figure 4A_scan_SIRT1 IP.tif]

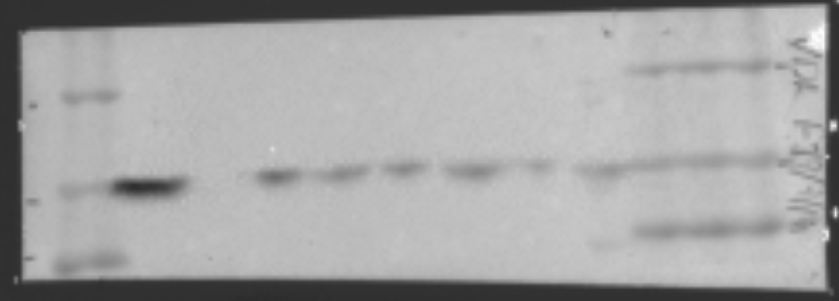

Supplement: Figure 4—source data 1. [file elife-86913-fig4-data1.zip › Fig4_source data/Fig.4A_raw data_pdf and TIFF/Figure 4A_scan_TBP.tif]

Figure 4B

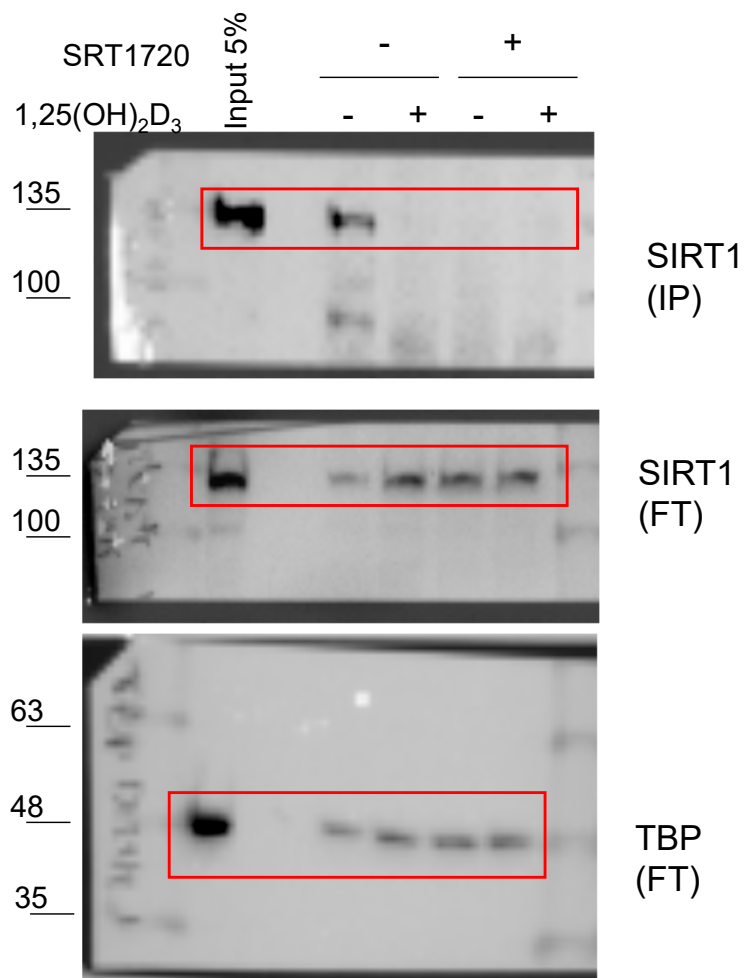

Supplement: Figure 4—source data 1. [file elife-86913-fig4-data1.zip › Fig4_source data/Fig.4B_raw data_pdf and TIFF/Fig.4B_raw data_pdf.pdf]

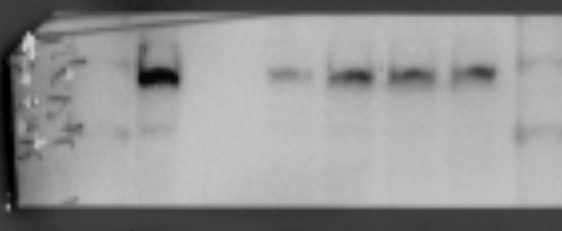

Supplement: Figure 4—source data 1. [file elife-86913-fig4-data1.zip › Fig4_source data/Fig.4B_raw data_pdf and TIFF/Figure 4B_scan_SIRT1 FT.tif]

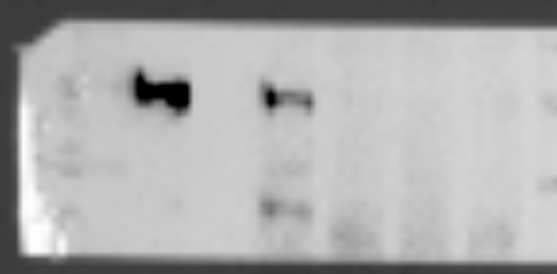

Supplement: Figure 4—source data 1. [file elife-86913-fig4-data1.zip › Fig4_source data/Fig.4B_raw data_pdf and TIFF/Figure 4B_scan_SIRT1 IP.tif]

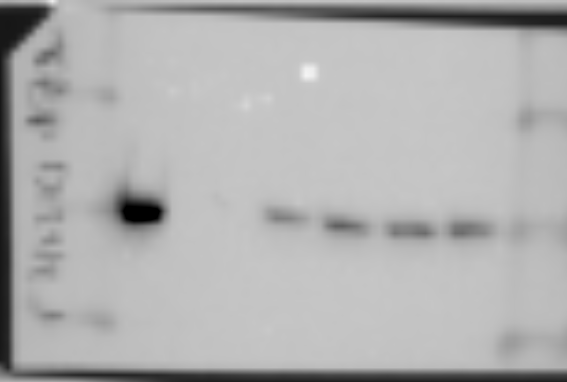

Supplement: Figure 4—source data 1. [file elife-86913-fig4-data1.zip › Fig4_source data/Fig.4B_raw data_pdf and TIFF/Figure 4B_scan_TBP FT.tif]

Figure 4C

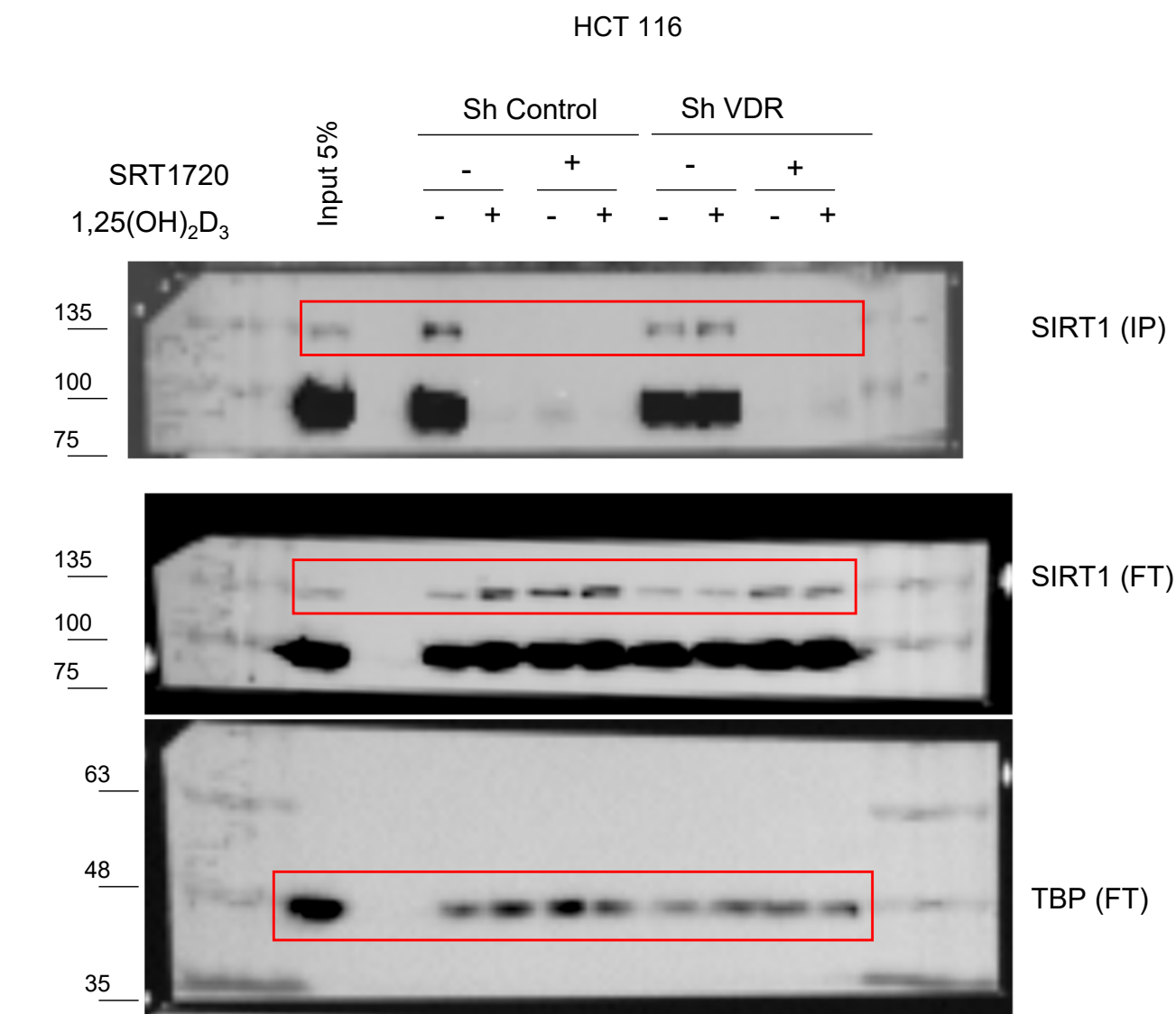

Supplement: Figure 4—source data 1. [file elife-86913-fig4-data1.zip › Fig4_source data/Fig.4C_raw data_pdf and TIFF/Fig.4C_raw data_pdf.pdf]

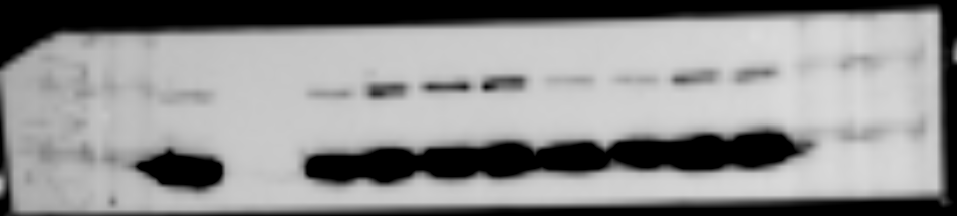

Supplement: Figure 4—source data 1. [file elife-86913-fig4-data1.zip › Fig4_source data/Fig.4C_raw data_pdf and TIFF/Figure 4C_scan_SIRT1 FT.tif]

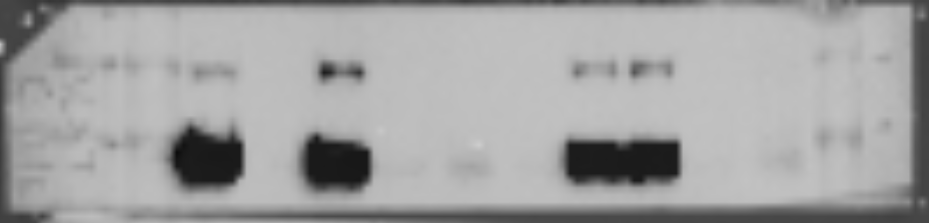

Supplement: Figure 4—source data 1. [file elife-86913-fig4-data1.zip › Fig4_source data/Fig.4C_raw data_pdf and TIFF/Figure 4C_scan_SIRT1 IP.tif]

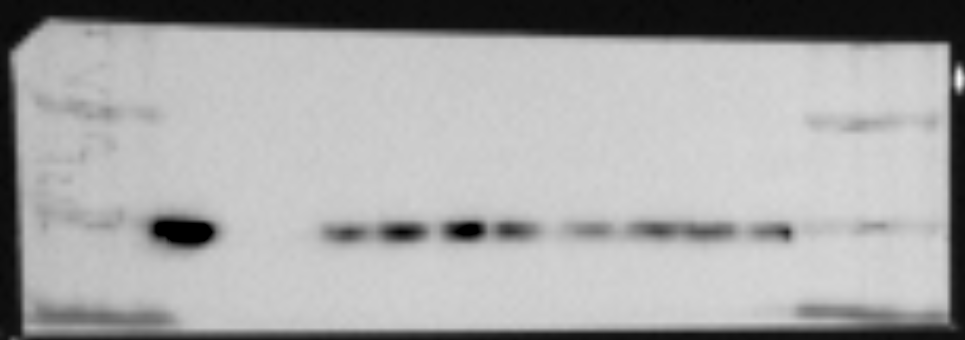

Supplement: Figure 4—source data 1. [file elife-86913-fig4-data1.zip › Fig4_source data/Fig.4C_raw data_pdf and TIFF/Figure 4C_scan_TBP FT.tif]

Figure 4E

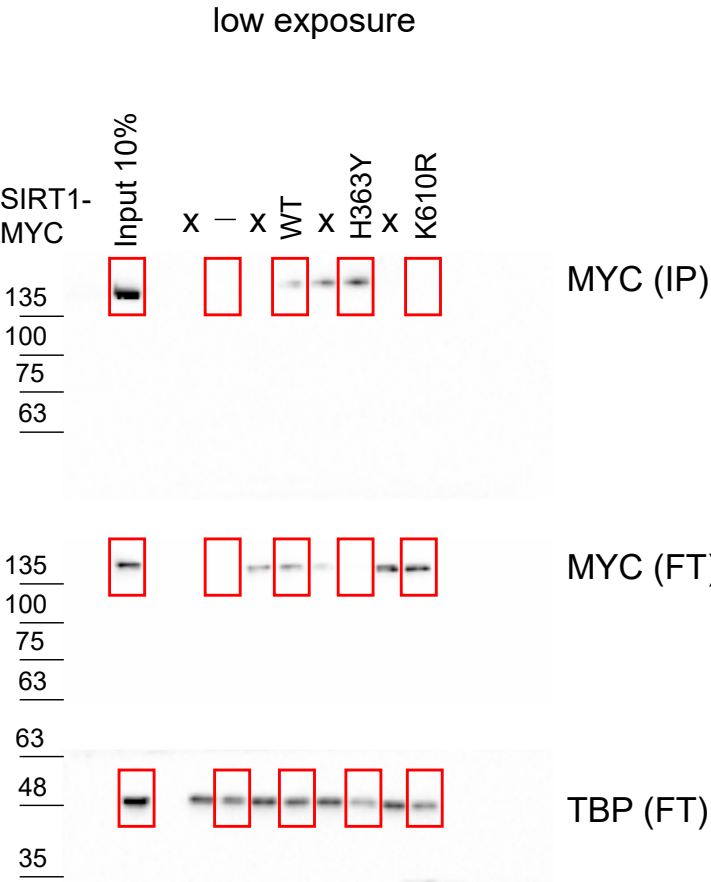

Supplement: Figure 4—source data 1. [file elife-86913-fig4-data1.zip › Fig4_source data/Fig.4E_raw data_pdf and TIFF/Fig.4E_raw data_pdf.pdf]

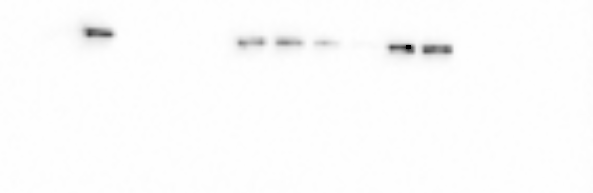

Supplement: Figure 4—source data 1. [file elife-86913-fig4-data1.zip › Fig4_source data/Fig.4E_raw data_pdf and TIFF/Figure 4E_scan_MYC FT.tif]

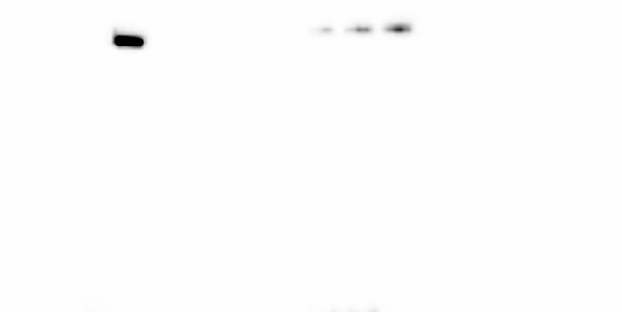

Supplement: Figure 4—source data 1. [file elife-86913-fig4-data1.zip › Fig4_source data/Fig.4E_raw data_pdf and TIFF/Figure 4E_scan_MYC IP.tif]

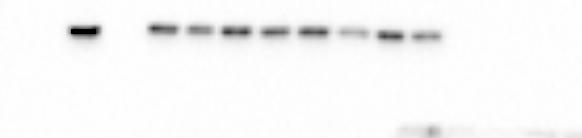

Supplement: Figure 4—source data 1. [file elife-86913-fig4-data1.zip › Fig4_source data/Fig.4E_raw data_pdf and TIFF/Figure 4E_scan_TBP_FT.tif]

Figure 4F

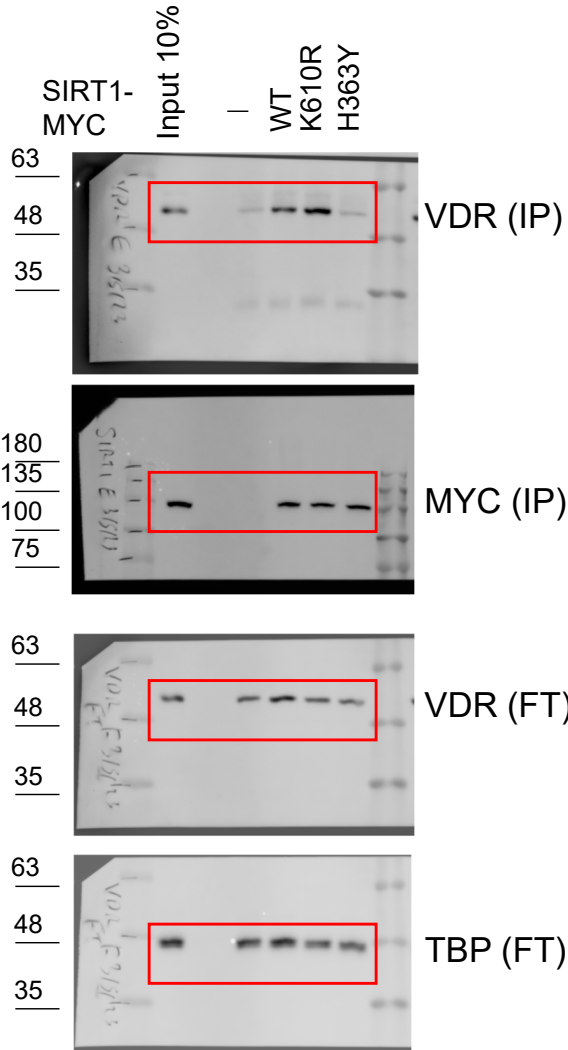

Supplement: Figure 4—source data 1. [file elife-86913-fig4-data1.zip › Fig4_source data/Fig.4F_raw data_pdf and TIFF/Fig.4F_raw data_pdf.pdf]

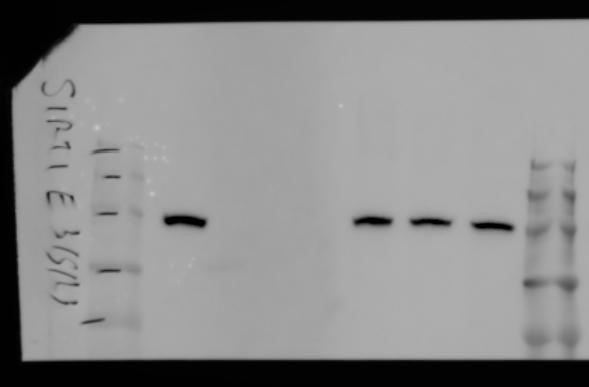

Supplement: Figure 4—source data 1. [file elife-86913-fig4-data1.zip › Fig4_source data/Fig.4F_raw data_pdf and TIFF/Figure 4F_scan_MYC IP.tif]

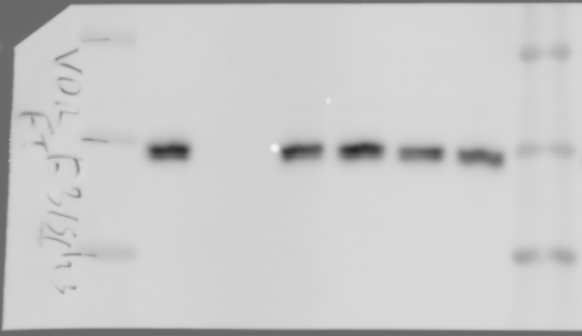

Supplement: Figure 4—source data 1. [file elife-86913-fig4-data1.zip › Fig4_source data/Fig.4F_raw data_pdf and TIFF/Figure 4F_scan_TBP FT.tif]

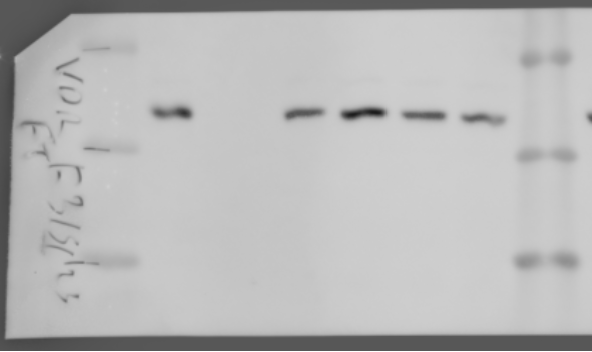

Supplement: Figure 4—source data 1. [file elife-86913-fig4-data1.zip › Fig4_source data/Fig.4F_raw data_pdf and TIFF/Figure 4F_scan_VDR FT.tif]

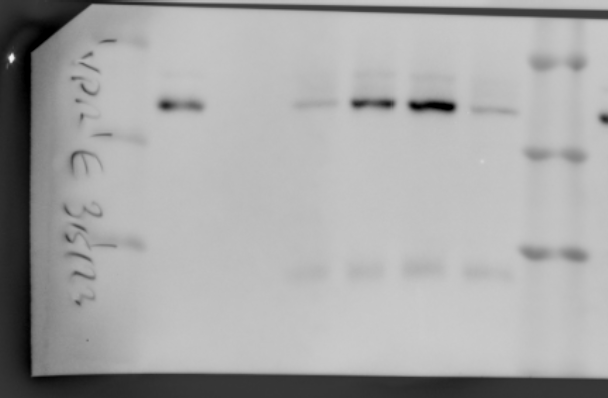

Supplement: Figure 4—source data 1. [file elife-86913-fig4-data1.zip › Fig4_source data/Fig.4F_raw data_pdf and TIFF/Figure 4F_scan_VDR IP.tif]

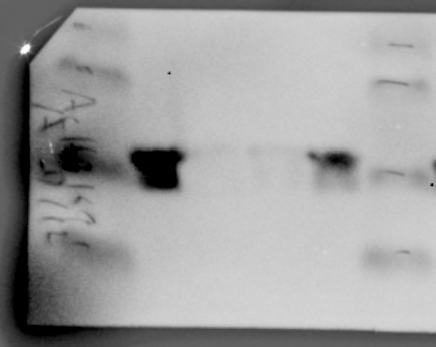

Supplement: Figure 4—source data 1. [file elife-86913-fig4-data1.zip › Fig4_source data/Fig.4G_raw data_pdf and TIFF/Figure 4G_scan_AceH3K9.tif]

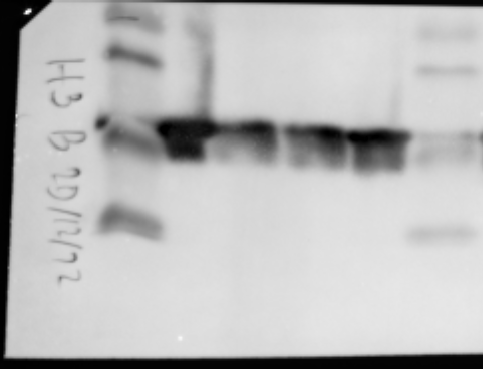

Supplement: Figure 4—source data 1. [file elife-86913-fig4-data1.zip › Fig4_source data/Fig.4G_raw data_pdf and TIFF/Figure 4G_scan_H3.tif]

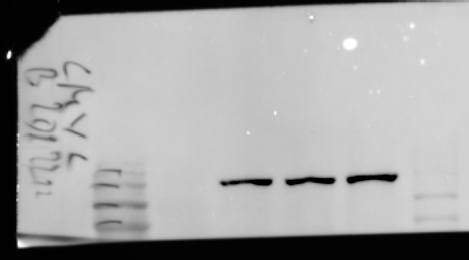

Supplement: Figure 4—source data 1. [file elife-86913-fig4-data1.zip › Fig4_source data/Fig.4G_raw data_pdf and TIFF/Figure 4G_scan_MYC.tif]

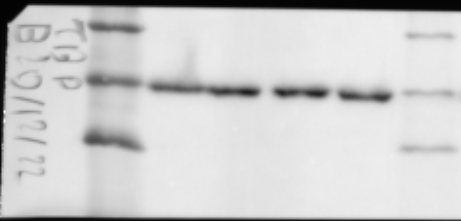

Supplement: Figure 4—source data 1. [file elife-86913-fig4-data1.zip › Fig4_source data/Fig.4G_raw data_pdf and TIFF/Figure 4G_scan_TBP.tif]

Figure 4G

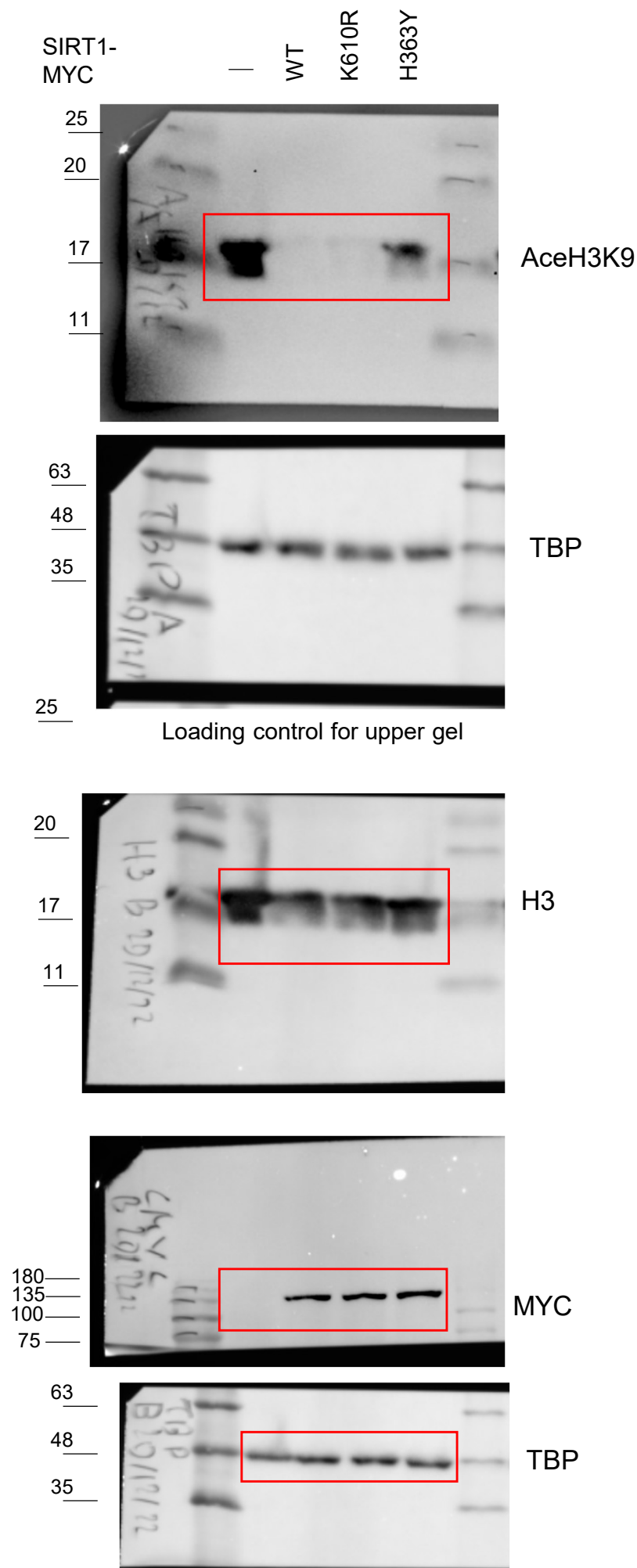

Supplement: Figure 4—source data 1. [file elife-86913-fig4-data1.zip › Fig4_source data/Fig.4G_raw data_pdf and TIFF/PDF Fig 4G.pdf]
